# Global Driving of Auroral Precipitation: 1. Balance of Sources

**DOI:** 10.1029/2022JA030323

**Published:** 2022-07-11

**Authors:** Agnit Mukhopadhyay, Daniel Welling, Michael Liemohn, Aaron Ridley, Meghan Burleigh, Chen Wu, Shasha Zou, Hyunju Connor, Elizabeth Vandegriff, Pauline Dredger, Gabor Tóth

**Affiliations:** ^1^ Climate and Space Sciences and Engineering Department University of Michigan Ann Arbor MI USA; ^2^ NASA Goddard Space Flight Center Greenbelt MD USA; ^3^ Department of Physics American University Washington DC USA; ^4^ Department of Physics University of Texas at Arlington Arlington TX USA; ^5^ Naval Research Laboratory Washington DC USA; ^6^ Department of Physics University of Alaska Fairbanks Fairbanks AK USA

**Keywords:** aurora, particle precipitation, ionospheric conductance, M‐I coupling, space weather, MHD modeling

## Abstract

The accurate determination of auroral precipitation in global models has remained a daunting and rather inexplicable obstacle. Understanding the calculation and balance of multiple sources that constitute the aurora, and their eventual conversion into ionospheric electrical conductance, is critical for improved prediction of space weather events. In this study, we present a semi‐physical global modeling approach that characterizes contributions by four types of precipitation—monoenergetic, broadband, electron, and ion diffuse—to ionospheric electrodynamics. The model uses a combination of adiabatic kinetic theory and loss parameters derived from historical energy flux patterns to estimate auroral precipitation from magnetohydrodynamic (MHD) quantities. It then converts them into ionospheric conductance that is used to compute the ionospheric feedback to the magnetosphere. The model has been employed to simulate the 5–7 April 2010 *Galaxy15* space weather event. Comparison of auroral fluxes show good agreement with observational data sets like NOAA‐DMSP and OVATION Prime. The study shows a dominant contribution by electron diffuse precipitation, accounting for ∼74% of the auroral energy flux. However, contributions by monoenergetic and broadband sources dominate during times of active upstream solar conditions, providing for up to 61% of the total hemispheric power. The study also finds a greater role played by broadband precipitation in ionospheric electrodynamics which accounts for ∼31% of the Pedersen conductance.

## Key Points

- A semi‐physical global modeling approach is used to estimate diffuse and discrete sources of auroral precipitation during the Galaxy15 event
- Diffuse sources contribute 74% of the total auroral power. Discrete sources are strongly driven by activity and can contribute up to 61%
- Broadband precipitation contributes 31% of the auroral Pedersen conductance playing a significant role in ionospheric electrodynamics

## 1 Introduction

High‐latitude precipitation of charged particles is a crucial driver of ionospheric electrodynamics (e.g., Kivelson & Russell, 1995). These particles precipitate from the near‐Earth plasma environment to form the aurora, and enhance the electrical conductance in the polar regions (e.g., Schunk & Nagy, 2009). Auroral precipitation is broadly defined into two types: diffuse and discrete aurora. Particles scattered into the loss cone by plasma waves create the diffuse aurora (Nishimura et al., 2020a and references therein). Diffuse particles precipitate into the upper atmosphere without the need of acceleration, and can consist of both electrons (e.g., Evans & Moore, 1979) and ions (e.g., Sergeev et al., 1983). Conversely, the discrete aurora is generated by particles that are accelerated into the ionosphere (e.g., Korth et al., 2014). These particles can be accelerated by geomagnetic field‐aligned electric fields (monoenergetic; e.g., Evans, 1974; Knight, 1973) or by dispersive Alfvén waves (broadband; e.g., Chaston et al., 2003; Ergun et al., 1998). The conductance enhancements caused by auroral precipitation are important to investigative studies of magnetosphere‐ionosphere coupling (e.g., Öztürk et al., 2020), since it regulates the closure of field‐aligned currents (FACs; Iijima & Potemra, 1976) and maintain the nonlinear feedback loop between the magnetosphere and the ionosphere (e.g., Merkine et al., 2003; Ridley et al., 2004). Since auroral currents are the dominant drivers of ground‐based magnetic perturbations in high‐latitudinal regions (e.g., Welling, 2019), auroral conductance is a crucial regulator of ground‐based space weather activity (Hartinger et al., 2017; Mukhopadhyay et al., 2020).

Despite their importance, the computation of auroral precipitation and derived conductances is not trivial in most global models. This is due to several reasons. First, the first‐principles‐driven calculation of conductance needs to account for ionosphere‐thermosphere dynamics like atmospheric chemistry and reaction rates (Yu et al., 2016). Most global models work around the complexity of ionosphere‐thermosphere dynamics by the use of empirical relationships like Robinson et al. (1987), Galand et al. (2001), and Kaeppler et al. (2015), that derive perpendicular conductances from precipitating fluxes. This method has limitations, as the empirical relations are based off of limited data set and have numerous associated uncertainties (Liemohn, 2020; Welling et al., 2017). Even so, most global models assume a two‐dimensional ionospheric domain (e.g., Goodman, 1995) which makes usage of an empirical conversion between fluxes and conductances undemanding. Recent work by Burleigh et al. (2019) has sought to incorporate a dedicated ionosphere‐thermosphere solver to incorporate realistic chemistry and altitudinal ionization rates to provide for a more accurate estimation of the conductance.

Second, estimating the kinetic description of particle precipitation is not straightforward in a global setup. This is especially challenging in magnetohydrodynamic (MHD) models which do not resolve pitch angle distributions and wave scattering. Fedder et al. (1995) expressed electron auroral energy and number fluxes as functions of MHD parameters using adiabatic kinetic theory. This work was further expanded by successive studies (Gilson et al., 2012; Raeder et al., 2001; Wiltberger et al., 2009; Yu et al., 2016, 2018; Zhang et al., 2015) who sought to improve the original methodology and incorporate multiple types of precipitation in the computation of ionospheric conductance in the aurora. Since adiabatic kinetic theory does not fully account for the kinetic physics of loss‐cone distributions, models like Ridley et al. (2004) and Mukhopadhyay et al. (2020) pursued a bypass, by using empirical relationships to derive precipitation with FACs. While this simplified the process of estimating auroral precipitation, the models only provide conductances in regions of high FACs and have statistical limits to physical phenomena like auroral expansion during extreme driving.

While the influence of auroral conductance on magnetospheric dynamics, ionospheric electrodynamics, and their coupled nonlinear feedback system is well known (e.g., Connor et al., 2016; Ebihara et al., 2005; Liemohn et al., 2005; Ozturk et al., 2017; Ridley et al., 2001; Welling & Ridley, 2010; Zheng et al., 2008), the contribution of each individual source of precipitation has not been widely studied, especially for variable solar wind driving. This is challenging to do with data, since most measurements of ionospheric conductance have significant underlying challenges and uncertainties (Ohtani et al., 2014). Empirical modeling efforts by Newell et al. (2009, 2014) have thoroughly studied the balance of auroral precipitation through the determination of multiple sources from in situ observations. Earlier studies by Hardy et al. (1985, 1989) and Brautigam et al. (1991) have sought to provide balance between different sources using upstream and/or space weather conditions. The comparison of observed FACs with in situ precipitation by Korth et al. (2014) provided further quantification of discrete sources of precipitation. Despite this, empirical approaches are limited by observational findings and lack the global perspective to relate drivers affecting auroral precipitation with quantities that they influence. Such a relationship can more easily be studied through a global first‐principles‐based modeling approach. Furthermore, with increasing usage of first‐principles‐based geospace models for operations‐grade space weather prediction (e.g., Cash et al., 2018; Pulkkinen et al., 2011, 2013; Rastätter et al., 2016), the need to quantify the impact of multiple sources of conductance on the M‐I feedback becomes ever more necessary.

This work describes the development of a novel modeling approach that predominantly uses a semiphysical method to estimate four sources of precipitation—monoenergetic, broadband, electron, and ion diffuse—using input variables from the Space Weather Modeling Framework (SWMF; Tóth et al., 2005, 2012). This model has been used to study salient aspects of the 5–7 April 2010 space weather event (e.g., Chen et al., 2015; Keesee et al., 2014) in order to determine the individual contributions of each source. In this endeavor, the article aims to address the following tasks: (a) quantification of individual contribution by each source of precipitation, (b) comprehension of the impact of upstream solar wind drivers on each source, and (c) discernment of the impact of each source on the net ionospheric conductance. Each task is addressed in Section 4 through the following comparative experiments:

1. Modeled auroral fluxes are validated against both patterns and hemispheric integrated quantities.
2. A case study is investigated that describes changes in each source of precipitation with changing upstream conditions.
3. Precipitation from each source is converted into ionospheric Hall and Pedersen conductances, and their contributions to both conductances are quantified.

## 2 Numerical Methodology

### 2.1 Geospace Numerical Setup of SWMF

**Figure 1:**
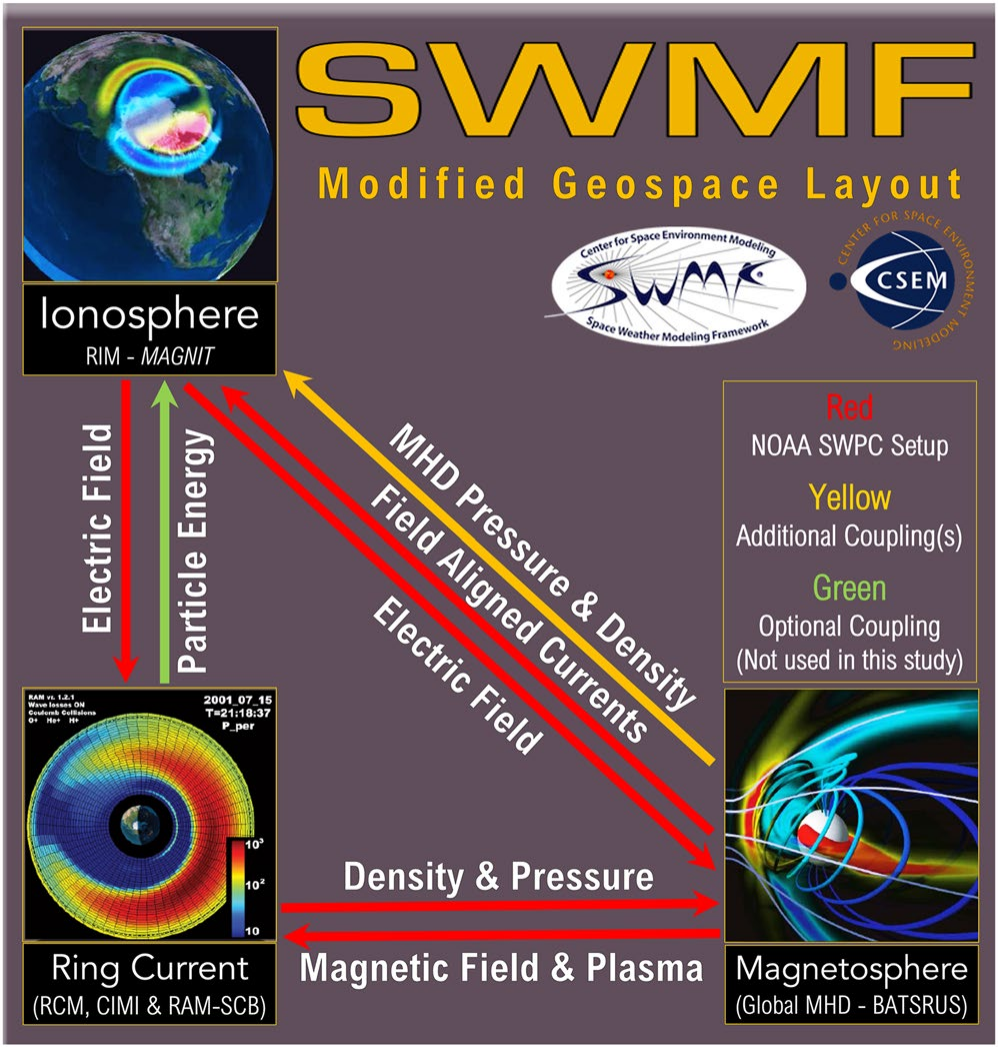
Numerical coupling between models within the geospace setup of Space Weather Modeling Framework (SWMF).

The geospace version of SWMF (see model layout in Figure 1), that is currently used for real‐time space weather prediction at NOAA‐SWPC (Cash et al., 2018), consists of three independent models that are numerically coupled together. The Block Adaptive Tree Solar‐Wind Roe Upwind Scheme (BATS‐R‐US; Gombosi et al., 2003; Powell et al., 1999) model uses ideal semirelativistic single‐fluid MHD equations to simulate the global magnetosphere. BATS‐R‐US’ magnetospheric domain is a three‐dimensional space in GSM coordinates. In the *x* axis, the domain spans 32 *R*_*E*_ on the dayside and 224 *R*_*E*_ on the nightside, while in the *y* and *z* coordinate axes, the domain spans 128 *R*_*E*_ in either directions. The model uses a block‐adaptive Cartesian grid to ensure highest spatial resolution in regions of interest. The grid resolution used in this study is similar to the *Hi‐Res SWPC* grid used in the study by Mukhopadhyay et al. (2020), details about which are found in Appendix A of Haiducek et al. (2017). To better capture energy‐dependent drift physics in the inner magnetosphere, BATS‐R‐US is coupled to a dedicated inner magnetosphere model (De Zeeuw et al., 2004). In the present setup, we have used the Rice Convection Model (RCM; e.g., Wolf et al., 1982) which solves for bounce‐averaged particle distribution in the ring current region. To drive this, RCM uses flux tube volumes from BATS‐R‐US and adjusts the MHD pressure and density in return.

SWMF also has a dedicated coupling to an ionospheric solver, the Ridley Ionosphere Model (RIM; e.g., Ridley et al., 2001), which computes ionospheric electrodynamics at an altitude of 110 km. RIM is a finite‐difference Poisson solver that computes the electrostatic potential and horizontal currents using FACs as input and a *prescribed* conductance pattern (Goodman, 1995). FACs are mapped down from near the inner boundary of BATS‐R‐US (typically between 3.5 *R*_*E*_ and 2.5 *R*_*E*_) to ionospheric altitudes (∼110 km). Additionally, BATS‐R‐US has optional settings to return plasma pressure and density values mapped near its inner boundary (De Zeeuw et al., 2004), which is then mapped onto the ionospheric grid (Yu et al., 2016). RIM returns the ionospheric potential to both BATS‐R‐US and RCM, which are subsequently used as inner boundary conditions.

Ridley et al. (2004) describes the computation of conductance in RIM. The solver estimates contributions by multiple sources like solar EUV illumination, auroral precipitation, and polar rain. Conductance due to EUV illumination affects the dayside, and is computed as a function of the solar zenith angle (Moen & Brekke, 1993). Enhancements due to starlight conductance and polar rain generally affect the nightside ionosphere, and are added in as constants. Conductance enhancements due to auroral precipitation are relatively complicated to estimate, since the aurora is driven by upstream driving conditions. In RIM, auroral conductance is typically computed by one of two dedicated models—Ridley Legacy Model (RLM; Ridley et al., 2004) or the Conductance Model for Extreme Events (CMEE; Mukhopadhyay et al., 2020). Both models are based off of assimilative maps (Ridley & Kihn, 2004), and use empirical relationships with FACs to estimate the auroral conductance (see Mukhopadhyay et al. (2020) for details).

### 2.2 The MAGNetosphere‐Ionosphere‐Thermosphere (MAGNIT) Auroral Precipitation Model

**Figure 2:**
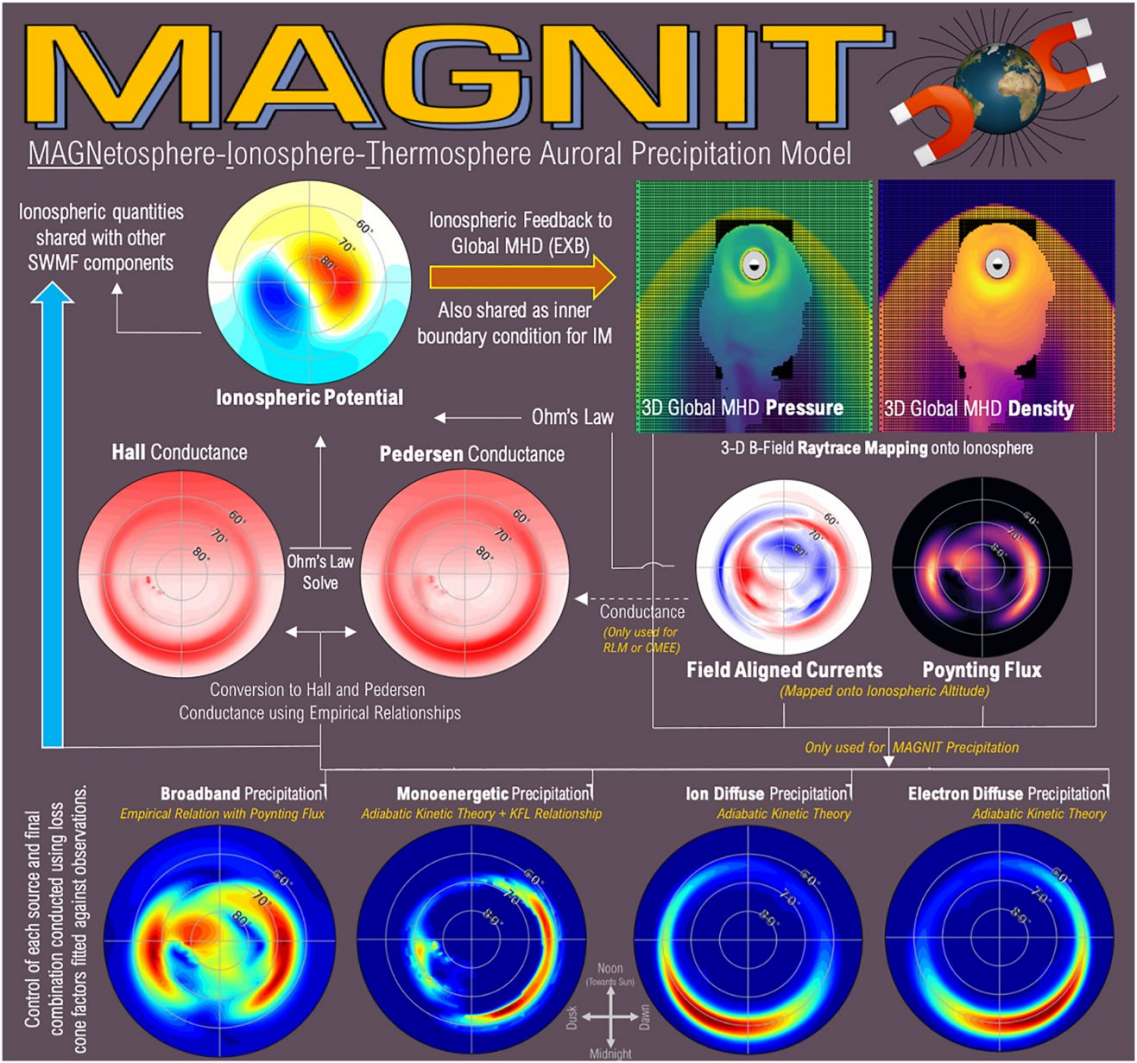
Schematic description of auroral conductance and subsequent ionospheric E‐field computation within Ridley Ionosphere Model (RIM) when equipped with MAGNetosphere‐Ionosphere‐Thermosphere (MAGNIT). Dotted lines indicate optional couplings when Ridley Legacy Model (RLM) or Conductance Model for Extreme Events (CMEE) is deployed. Note: Color bar scale is not uniform across dial plots.

In this study, we introduce the MAGNetosphere‐Ionosphere‐Thermosphere (MAGNIT) auroral model that computes individual sources of precipitation in SWMF. MAGNIT culminates from a series of modeling developments within RIM (Burleigh et al., 2019; Liemohn et al., 2018; Mukhopadhyay et al., 2018, 2019, 2021a) that replace the existing empirical conductance models with state‐of‐the‐art numerical couplers and solvers to estimate auroral dynamics. Figure 2 describes the ionospheric setup of MAGNIT within RIM. Advanced numerical couplings are introduced to transfer field line‐traced values of bulk quantities like pressure and density from BATS‐R‐US to RIM, mapping the values down from the inner boundary of MHD to 110‐km altitude. MAGNIT uses adiabatic kinetic theory to compute auroral fluxes from the MHD state variables (e.g., Fedder et al., 1995). This investigation uses MAGNIT to estimate four different sources of precipitation—electron diffuse, ion diffuse, monoenergetic, and broadband—and quantify their individual contributions. The computation of each source is described in the following.

#### 2.2.1 Electron and Ion Diffuse Precipitation

Parameterization of diffuse fluxes in MAGNIT observes the following formulation:

(1) $$\Phi_{N_{\text{diff}\left(e,i\right)}}=\alpha_{1}\left(e,i\right)\times \frac{N_{e,i}T_{e,i}^{1/2}}{\sqrt{2\pi m_{e,i}}}$$

(2) $$\Phi_{E_{\text{diff}\left(e,i\right)}}=\alpha_{2}\left(e,i\right)\times \frac{2N_{e,i}T_{e,i}^{3/2}}{\sqrt{2\pi m_{e,i}}}$$

Here, $\Phi_{N_{\text{diff}}}$ and $\Phi_{E_{\text{diff}}}$ are the diffuse number and energy fluxes, respectively, *N* is the particle number density, *T* is the particle thermal temperature (derived from MHD), and *m* is the particle mass. The equations have been denoted for both electrons and ions by subscripts *e* and *i*, respectively. The multipliers *α*_1_(*e*, *i*) and *α*_2_(*e*, *i*) act as the particle filling rate of the loss cone, and are optimized using data‐model comparisons (discussed in more detail in Section 3). This is necessary because global MHD is unable to resolve pitch angle distributions, and therefore cannot accurately predict particle loss from the magnetosphere without further parameterization (e.g., Zhang et al., 2015). The remaining fractions are the standard solution for total particle and energy flux contained in a Maxwellian distribution for all pitch angles (e.g., Gombosi, 1994).

*T*_*e*_ is assumed to be 1/6 of the single‐fluid MHD temperature, *T*_MHD_, based on an electron‐to‐ion temperature ratio of 1:5, as observed in the plasma sheet (Fedder et al., 1995; Paschmann et al., 1993; Phan et al., 1994). This is a simplistic approximation however; though this relationship is typically valid for low‐energy plasma sheet particles in the near‐Earth region (Wang et al., 2012), a more accurate representation between the electron and ion temperature is planned in future modeling developments. This will incorporate the independent computation of electron plasma temperature using a two‐fluid or multifluid MHD approach (e.g., Glocer et al., 2009, 2020) to work around this approximation. Unlike some modeling approaches (e.g., Zhang et al., 2015), the precipitation pattern of either diffuse sources do not undergo any spatial variations. This is done in order to better retain dynamic changes in auroral boundaries across activity levels. Ion precipitation occurs in regions where the field line curvature becomes comparable to the particle gyroradius (Sergeev & Tsyganenko, 1982; Sergeev et al., 1983). To simulate this, we have used a modified version of the model developed by Gilson et al. (2012), where a step function is used as a function of the *κ*‐parameter (e.g., Büchner & Zelenyi, 1987). In lieu of a field line‐estimated *κ*, an equatorward and poleward boundary based on the peak strength of field line‐traced pressure was used to define the bounds of the step function.

After the computation of the ion and electron fluxes, the average energy ${\bar{E}}_{\text{diff}\left(e,i\right)}$ is computed as $\Phi_{E_{\text{diff}\left(e,i\right)}}/\Phi_{N_{\text{diff}\left(e,i\right)}}$. Using the empirical relationships developed by Robinson et al. (1987), the average energy and energy flux for the electron diffuse source are converted into Hall and Pedersen conductances. Similarly, MAGNIT uses the ion flux and energies to compute the ion‐driven conductance via the Galand and Richmond (2001) empirical relationships.

#### 2.2.2 Monoenergetic Precipitation

MAGNIT estimates monoenergetic precipitation using the Knight‐Fridman‐Lemaire (KFL; Fridman & Lemaire, 1980; Knight, 1973; Lyons et al., 1979) relationship that estimates electrons accelerated by a quasi‐static parallel potential drop along a magnetic field line. We broaden our assumption of an isotropic Maxwellian particle distribution by estimating incident electrons upon a field‐aligned potential drop *V*. Using this modification, we follow the KFL procedure to estimate the first and third moments of the distribution to compute the downward number and energy flux (e.g., Yu et al., 2016; Zhang et al., 2015)

(3) $$\Phi_{N_{\text{mono}}}=\alpha_{3}\times \frac{N_{e}T_{e}^{1/2}}{\sqrt{2\pi m_{e}}}\left[\frac{B_{\mathrm{iono}}}{B_{ps}}-\left(\frac{B_{\mathrm{iono}}}{B_{ps}}-1\right)e^{-\frac{q_{e}VB_{ps}}{T_{e}\left(B_{\mathrm{iono}}-B_{ps}\right)}}\right]$$

(4) $$\Phi_{E_{\text{mono}}}=\alpha_{4}\times \frac{2N_{e}T_{e}^{1/2}}{\sqrt{2\pi m_{e}}}\left[\frac{1-e^{-\frac{q_{e}VB_{ps}}{T_{e}\left(B_{\mathrm{iono}}-B_{ps}\right)}}}{2\left(1+\left(\frac{B_{\mathrm{iono}}-B_{ps}}{B_{\mathrm{iono}}}\right)e^{-\frac{q_{e}VB_{ps}}{T_{e}\left(B_{\mathrm{iono}}-B_{ps}\right)}}\right)}q_{e}V+T_{e}\right]\times \left[\frac{B_{\mathrm{iono}}}{B_{ps}}-\left(\frac{B_{\mathrm{iono}}}{B_{ps}}-1\right)e^{-\frac{q_{e}VB_{ps}}{T_{e}\left(B_{\mathrm{iono}}-B_{ps}\right)}}\right]$$

Similar to Equations 1 and 2, $\Phi_{N_{\text{mono}}}$ and $\Phi_{E_{\text{mono}}}$ stand for the monoenergetic number and energy fluxes, respectively, *N*_*e*_ is the electron number density, *T*_*e*_ is the electron temperature in the nightside plasma sheet, *m*_*e*_ is electron mass, and *q*_*e*_ is the elementary charge. *B*_*iono*_ and *B*_*ps*_ signify the magnetic field strengths at ionospheric altitude (assumed at 110 km in the present model) and at the source region, respectively; their ratio *B*_*iono*_/*B*_*ps*_ is the magnetic mirror ratio. The source region in our simulations is assumed to be the plasma sheet (Yu et al., 2016) and the mirror ratio is assumed to be dipolar. The multipliers *α*_3_ and *α*_4_ are parameters that represent the degree of loss‐cone filling in the electron source region. Unspecified by the MHD fluid approach, we change these factors to scale the resulting fluxes.

In regions of upward FAC (*J*_‖_), assuming that the current is entirely carried by electrons, the number flux of monoenergetic precipitation can be expressed as

(5) $$\Phi_{N_{\text{mono}}}=J_{\|}/q_{e}$$

Therefore, the potential drop *V* can be expressed in terms of *J*_‖_ as

(6) $$V=\frac{T_{e}\left(B_{ps}-B_{\mathrm{iono}}\right)}{q_{e}B_{ps}}\ln\left(\frac{B_{\mathrm{iono}}-B_{ps}\frac{J_{\|}}{q_{e}}\frac{\sqrt{2\pi m_{e}}}{\alpha_{3}N_{e}T_{e}^{1/2}}}{B_{\mathrm{iono}}-B_{ps}}\right)$$

The above condition is only valid for the logarithm not being zero, which indicates that

(7) $$B_{ps}\leq B_{ps}\frac{J_{\|}}{q_{e}}\frac{\sqrt{2\pi m_{e}}}{\alpha_{3}N_{e}T_{e}^{1/2}}\leq B_{\mathrm{iono}}$$

must be satisfied for a monoenergetic source of precipitation. Also, the potential structure along the field line must satisfy the relationships described by Chiu and Schulz (1978), in which case a more complicated approach is needed (e.g., Liemohn & Khazanov, 1998). Fortunately, this influence is small for potential drops accelerating electron auroral precipitation (e.g., Khazanov et al., 1998). In the absence of parallel fields (*V* = 0), the above equations are reduced to Equations 1 and 2 that model the electron diffuse precipitation. Similar to diffuse precipitation, the average energy for monoenergetic precipitation ${\bar{E}}_{\text{mono}}$ is computed as $\Phi_{E_{\text{mono}}}/\Phi_{N_{\text{mono}}}$. Using the empirical relation by Robinson et al. (1987), the Hall and Pedersen conductances are computed and combined with the diffuse sources using a vector sum.

#### 2.2.3 Broadband Precipitation

Broadband precipitation is driven by low‐energy electrons that are accelerated by dispersive Alfvén waves (e.g., Ergun et al., 1998). Successive investigations (Chaston et al., 2003; Strangeway, 2010; Zhang et al., 2014, 2015) have characterized a relationship between broadband flux and the Alfvénic Poynting flux. In MAGNIT, we use a similar approach as in Zhang et al. (2015), to estimate the broadband number and electron flux as an empirical function of the Poynting flux. The relation is shown as

(8) $$\Phi_{N_{\text{bbnd}}}=3\times 10^{9}\times {\left(\alpha_{5}\times S_{\|}\right)}^{0.47}$$

(9) $$\Phi_{E_{\text{bbnd}}}=2\times {\left(\alpha_{6}\times S_{\|}\right)}^{0.5}$$

Similar to previous sources, $\Phi_{N_{\text{bbnd}}}$ and $\Phi_{E_{\text{bbnd}}}$ stand for the broadband number and energy fluxes, respectively, which are collectively used to define the broadband average energy <*E*_bbnd_> as $\Phi_{E_{\text{bbnd}}}/\Phi_{N_{\text{bbnd}}}$. *S*_‖_ is the Poynting flux into the ionosphere. Unlike the model by Zhang et al. (2015), MAGNIT does not use AC Poynting flux which is a more direct measure of small‐scale Alfvénic energy, but instead relies on DC Poynting flux (Yu et al., 2010) derived from Joule heating in the ionosphere (e.g., Rastätter et al., 2016). Due to the empirical nature of the above equations, the multipliers *α*_5_ and *α*_6_ act as empirical moderators of the Poynting energy, since DC Poynting flux typically has higher values than electron precipitation (Janhunen et al., 2005). Broadband fluxes are converted into electron‐driven conductances by the Robinson et al. (1987) relationship.

Following Zhang et al. (2015), the broadband contribution is added linearly to the total conductance due to it enhancing ion density in the bottomside *F* region rather than the *E* region of the ionosphere. Since the rest of the sources are added as a vector sum (Wallis & Budzinski, 1981), the final sum of auroral sources to ionospheric conductance is computed as follows:

(10) $$\Sigma_{\text{Aurora}}=\sqrt{\Sigma_{e^{-}\text{diff}}^{2}+\Sigma_{i^{+}\text{diff}}^{2}+\Sigma_{\text{mono}}^{2}}+\Sigma_{\text{bbnd}}$$

where Σ_Aurora_ stands for the total auroral conductance (Hall or Pedersen) $\Sigma_{e^{-}\;\text{diff}}$, $\Sigma_{i^{+}\;\text{diff}}$, Σ_mono_, and Σ_bbnd_ stand for Hall or Pedersen conductances from the electron diffuse, ion, monoenergetic, and broadband sources, respectively.

### 2.3 Comparisons Against Observations and Empirical Models

In order to quantify the contribution by each source to the net auroral flux and energies, the hemispheric power (integrated energy flux over a hemisphere), hemispheric number flux (integrated number flux over a hemisphere), and overall average energy (hemispheric power/hemispheric number flux) were computed. Percent contributions by each source of precipitation (%C _source_) were defined as

(11) $$\%C_{\;\text{source}}=\frac{Q_{\text{source}}}{Q_{\text{aurora}}}$$

where *Q*_source_ stands for a given quantity from an individual source and *Q*_aurora_ stands for the same quantity from all auroral sources combined, with quantity *Q* being either hemispheric power or hemispheric number flux. Determining the contribution to ionospheric conductance is not as straightforward as in the case of energy flux and number flux. This is because the broadband source of conductance is added linearly to the total conductance. Therefore, contribution to the conductance has been defined in two ways—the total contribution, which computes the percentage contribution of a source as a fraction of the linear sum of conductances (similar to Equation 11), and the resultant contribution, which computes the percentage contribution for the sources in the following way:

(12) $$\%{\;C}_{\;\text{source}}=\frac{\Sigma_{\text{source}}^{2}}{\Sigma_{\text{aurora}}^{2}}\;\text{where}\;\text{source}\;=\;e^{-}\;Di\mathrm{ff},\;i^{+}\;Di\mathrm{ff},\;Mono$$

and

(13) $$\%{\;C}_{\;\text{broadband}}=1-\frac{\left(\Sigma_{e^{-}\text{Diff}}^{2}+\Sigma_{i^{+}\text{Diff}}^{2}+\Sigma_{\text{Mono}}^{2}\right)}{\Sigma_{\text{aurora}}^{2}}$$

where *e* ^*−*^Diff, *i* ^+^Diff, and Mono indicate electron diffuse, ion diffuse, and monoenergetic sources, respectively, and Σ is the Hall or Pedersen conductance.

Modeled results of auroral fluxes have been evaluated through comparisons against observations and multiple derived estimates. The study uses hemispheric power data from in situ observations measured by the Defense Meteorological Satellite Program (DMSP) and National Oceanic and Atmospheric Administration (NOAA) satellites (Emery et al., 2006, 2008). The observations are hourly averaged and span the past ∼30 years (1978–2013 for electrons; 1983–2013 for ions). The study uses the empirical models, OVATION Prime (Newell et al., 2009; shortened to OV Prime) and the AE‐driven Feature Tracking of Aurora (FTA; Wu et al., 2021) for comparison of energy flux and average energies in the auroral region. OV Prime is developed from multiple observations from DMSP during the years 1988–1998, while FTA is based on 1.5 years of Polar Ultraviolet Imager data. Both OV Prime and MAGNIT account for multiple (and similar) sources of precipitation. This has enabled us to compare individual contributions to hemispheric power, number flux, and energies in this study. Multiple empirical functions relating hemispheric power and number flux to space weather indices, auroral electrojet parameters, and/or upstream conditions exist. While the usage of all such models is not possible, this study identified and employed five empirical models—Brautigam et al. (1991), Ahn et al. (1983), Lu et al. (1998), Østgaard et al. (2002), and Korth et al. (2014)—to compare modeled predictions. Of these, the first four models provide total hemispheric power estimates—the model by Brautigam et al. (1991) estimates fluxes for electrons and ions separately, and is driven using solar wind inputs; remaining models are driven using AE/AU/AL values, and seek to establish a relationship between the energy deposition by electron precipitation and geomagnetic indices (see Østgaard et al. (2002) for detailed comparisons). The model by Korth et al. (2014) estimates discrete energy fluxes using upward FACs, and was predominantly used to validate modeled monoenergetic and broadband precipitation.

Additionally, simulated cross‐polar cap potential (CPCP), integrated FACs (iFACs; Anderson et al., 2017), *Kp* and Sym‐H were also compared against observations. CPCP values were compared against derived estimates obtained from the AMIE model and the Super Dual Auroral Radar Network (SuperDARN; e.g., Khachikjan et al., 2008). Observations for *Kp* and Sym‐H were obtained from the Kyoto Observatory, while iFAC observations were acquired from the Active Magnetosphere and Planetary Electrodynamics Response Experiment (AMPERE) mission (Anderson et al., 2014; Waters et al., 2020).

Evaluation of modeled results against observations has been quantified using two metrics—median absolute percentage error (MAPE) and the exclusion parameter (EP). MAPE provides an absolute value of the relative percentage error, and has been commonly used as a measure of accuracy of prediction (e.g., Morley et al., 2018). EP measures the accuracy of prediction against multiple observation‐derived estimates, by accounting for all data that is outside the range of observed values (Mukhopadhyay et al., 2021b). Low values of both metrics are generally assumed as a good prediction (e.g., Liemohn et al., 2021).

## 3 Parameterization of Sources

Due to its inability to compute loss‐cone distributions, MAGNIT uses flux multipliers *α*_*s*_ (for each source *s*) to regulate the final value of energy and number flux. A comparative study of modeled results to OV Prime and NOAA‐DMSP was undertaken to determine *α*_*s*_. Initially, a similar procedure to Zhang et al. (2015) was applied—hemispheric fluxes from each auroral source were compared against OV Prime estimates to initialize *α*_*s*_ for each source. Both SWMF and OV Prime were run for diverse driving conditions—Table 1 lists idealized solar wind conditions used for these simulations. For each SWMF run, the magnetosphere was preconditioned for 6 hr by first driving with southward IMF *B*_*z*_ = −5 nT for 3 hr, followed by northward IMF *B*_*z*_ = +5 nT for 3 hr. Post preconditioning, the simulation was driven for 3 more hr with the values listed in Table 1. The solar wind velocity, number density, and temperature in these runs were kept the same as Zhang et al. (2015) for consistency.

**Table 1:** IMF Conditions Used in Each Test Run to Determine *α*_*s*_

Run	*B*_(*x*,*y*,*z*)_ (nT)	$\frac{d\Phi_{\text{MP}}}{dt}$
A	(0, 0, −1)	0.29e + 04
B	(0, 0, −2)	0.47e + 04
C	(0, 0, −3)	0.61e + 04
D	(0, 0, −4)	0.74e + 04
E	(0, 0, −5)	0.86e + 04
F	(0, 0, −6)	0.97e + 04
G	(0, 0, −7)	1.08e + 04
H	(0, 0, −8)	1.18e + 04
I	(0, 0, −9)	1.28e + 04
J	(0, 0, −10)	1.37e + 04
K	(0, 0, −11)	1.46e + 04
L	(0, 0, −12)	1.54e + 04
M	(0, 0, −13)	1.63e + 04
N	(0, 0, −14)	1.71e + 04
O	(0, 0, −15)	1.79e + 04

*Note*. A solar wind velocity of 400 km/s in the *x*‐direction and a number density of 5 cm^−3^ were used in each test run. The third column shows the Newell coupling function, *d*Φ_MP_/*dt* = $v^{4/3}{\left(B_{x}^{2}+B_{y}^{2}\right)}^{1/3}\sin^{8/3}\theta_{C}$, where *θ*_*C*_ is the IMF clock angle, is listed in the fifth column.

**Figure 3:**
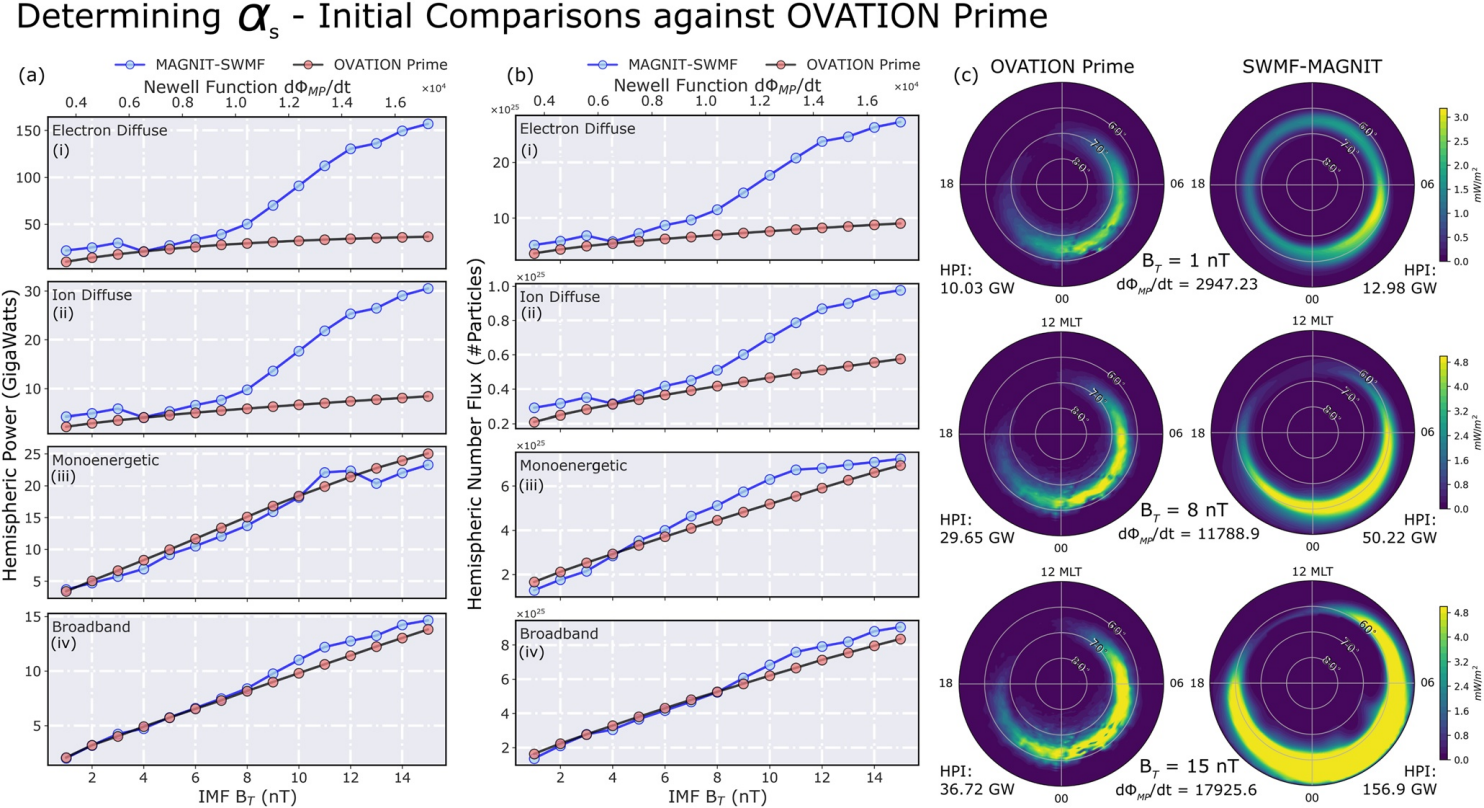
Initial determination of *α*_*s*_ by comparing MAGNetosphere‐Ionosphere‐Thermosphere (MAGNIT) runs against OVATION Prime values. Comparison of (a) hemispheric power and (b) total hemispheric number flux for (i) electron diffuse, (ii) ion diffuse, (iii) monoenergetic, and (iv) broadband precipitation through varying driving conditions. Here, IMF *B*_*T*_ = $\sqrt{B_{y}^{2}+B_{z}^{2}}$. (c) Dial‐plot comparison of diffuse electron precipitation from both models for (row‐wise) Runs A, H, and O, indicating change in morphology and auroral strength with increasing activity.

Figures 3a and 3b compare hemispheric power and number flux predictions, respectively, from MAGNIT against OV Prime values. Each subplot corresponds to a distinct source. The *x* axis in each subplot displays the variation in driving conditions, using the Newell function *d*Φ_MP_/*dt* (Newell et al., 2007) and IMF *B*_*z*_. *α*_*s*_ for each source was defined using OV Prime fluxes (*Q*_OP_) and MAGNIT fluxes (*Q*_MAGNIT_) as the median of their ratio, i.e., *α*_*s*_ = median(*Q*_OP_/*Q*_MAGNIT_). The final values of MAGNIT‐estimated precipitation (denoted in blue) were scaled by multiplication with the initialized values of *α*_*s*_, for comparison against OV Prime estimates (in black‐red). In parts a(iii–iv) and b(iii–iv) of Figure 3, monoenergetic and broadband fluxes show good agreement with OV Prime values when scaled. However, for both diffuse sources (parts a(i–ii) and b(i–ii) in Figure 3), MAGNIT estimates are much higher than OV Prime, and the two sets of prediction diverge from each other beyond an IMF *B*_*z*_ value of −6 nT. The difference in diffuse fluxes is further elucidated in Figure 3c, where dial plots of electron diffuse energy flux from OV Prime (left) and MAGNIT (right) are compared for Runs A, H, and O (see Table 1). With increasing activity, MAGNIT displays stronger auroral precipitation and significant expansion in the auroral oval. By contrast, OVATION Prime caps the max value of the energy flux, and displays minute expansion of the auroral oval. OV Prime has been shown to underestimate hemispheric fluxes during extreme driving (Newell et al., 2014). The oval expansion is an aspect of MAGNIT's usage of MHD pressure to compute diffuse fluxes (see Section 5 for further details).

**Figure 4:**
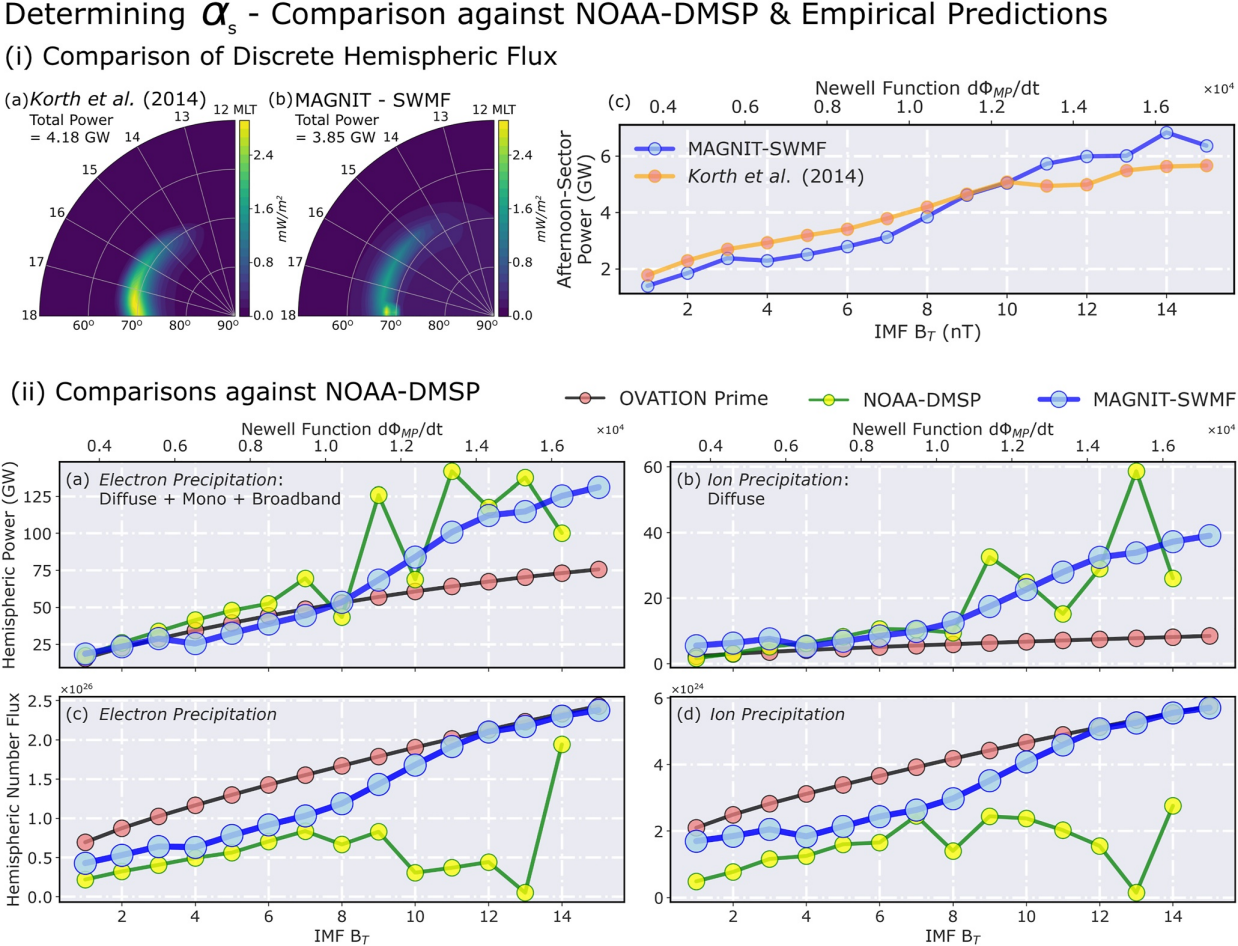
Finalization of *α*_*s*_ values by comparing MAGNetosphere‐Ionosphere‐Thermosphere (MAGNIT) runs against in situ observations and empirical predictions. (i) Comparison of discrete HP—(left) postnoon quarter‐dial plots of (a) Korth et al. (2014) and (b) MAGNIT at IMF *B*_*T*_ = 8 nT, (right) quarter‐hemispheric power across variable driving conditions for same models. (ii) Comparison against NOAA‐DMSP HP—comparison of hemispheric power for (a) electron and (b) ion power, and (c)–(d) their respective number fluxes compared against NOAA‐DMSP and OV Prime estimates over varying driving conditions.

To verify diffuse contributions, the comparative analysis was expanded to include hourly averaged hemispheric power observed by the NOAA‐DMSP satellite chain over the past >30 years (1978–2013) (Emery et al., 2006, 2008). This is, in essence, an extension to the comparison against OV Prime, since OV Prime itself is based on multiple observations from DMSP. Unlike OV Prime, the NOAA‐DMSP observations do not distinguish between different precipitative sources, instead informing only of contributions made by electrons and ions to the total energy flux, respectively. Therefore, the extended comparisons were conducted in two phases—(a) verifying discrete contributions against Korth et al. (2014) and (b) comparing total electron and ion fluxes against NOAA‐DMSP. The empirical relationship given in Korth et al. (2014) relates upward FACs in the dusk‐afternoon sector with discrete (mono + broadband) energy flux. Comparisons of discrete hemispheric power in the dusk‐afternoon sector for both models are shown in Figure 4i. The *Korth14* model was driven using FAC estimates from each SWMF run. MAGNIT results display reasonable agreement with the *Korth14* model in subplot 4(i‐c) when scaled using the *α*_*s*_ values deduced from Figure 3. Values for *α*_*s*_ are determined as the median of observed‐to‐modeled ratio for each source. In subplot 4(i‐c), these values were determined for monoenergetic and broadband sources separately to deduce the total discrete contribution. Comparison of model‐model energy flux distributions in the dusk‐afternoon sector (parts i‐a and i‐b of Figure 4) also exhibit similarities in pattern.

Figure 4ii shows final comparisons between NOAA‐DMSP observations (in green) and MAGNIT estimates (in blue) over varying IMF *B*_*z*_. The values from OV Prime (in black‐red) have also been plotted for clarity. The NOAA‐DMSP data set was binned by the Newell function (*d*Φ_MP_/*dt*) to provide median values for each bin. Since the in situ measurements do not observe number flux of precipitating particles, they were derived using average energies of electrons and ions from Hardy et al. (1985, 1989). *α*_*s*_ values for both electron and ion diffuse precipitation were adjusted based on the median of ratio between NOAA‐DMSP and MAGNIT fluxes, and between OV Prime and MAGNIT fluxes. Scaled comparisons of MAGNIT fluxes in Figures 4ii‐a and 4ii‐b show reasonable agreement with NOAA‐DMSP hemispheric power values during extreme driving for both electrons and ions. However, in Figures 4ii‐c and 4ii‐d, NOAA‐DMSP estimates lower number fluxes for stronger driving conditions, during which MAGNIT fluxes match well with OV Prime values.

The finalized values of *α*_*s*_ (and their associated uncertainties) are presented in Table 2. The associated uncertainty is derived using the standard deviation in *α*_*s*_ from each run, while the median value has been used as the finalized value for simulations. The uncertainty in *α*_*s*_ is bound to impact the final modeling results, which would have far‐reaching impacts on our space weather results. Investigation of this impact is beyond the scope of the present study, and will be researched in further detail in a future study. The finalized values were retrofitted into MAGNIT to balance each source of precipitation during run‐time. This procedure has been applied for the simulation of the *Galaxy15* event described in Section 4.

**Table 2:** Finalized Value of *α*_*s*_ for Auroral Number and Energy Flux Determined for Each Precipitative Source

Source	*α*_*s*_ (NumFlux)		*α*_*s*_ (EFlux)	
	Variable	Value	Variable	Value
Electron diffuse	*α*_1,*e*_	0.055 ± 27.4%	*α*_2,*e*_	0.224 ± 18.3%
Ion diffuse	*α*_1,*i*_	0.038 ± 13.8%	*α*_2,*i*_	0.207 ± 23.8%
Monoenergetic	*α*_3_	0.741 ± 4.48%	*α*_4_	0.995 ± 4.68%
Broadband	*α*_5_	0.244 ± 4.70%	*α*_6_	2.247 ± 5.12%

## 4 Event Simulation

**Figure 5:**
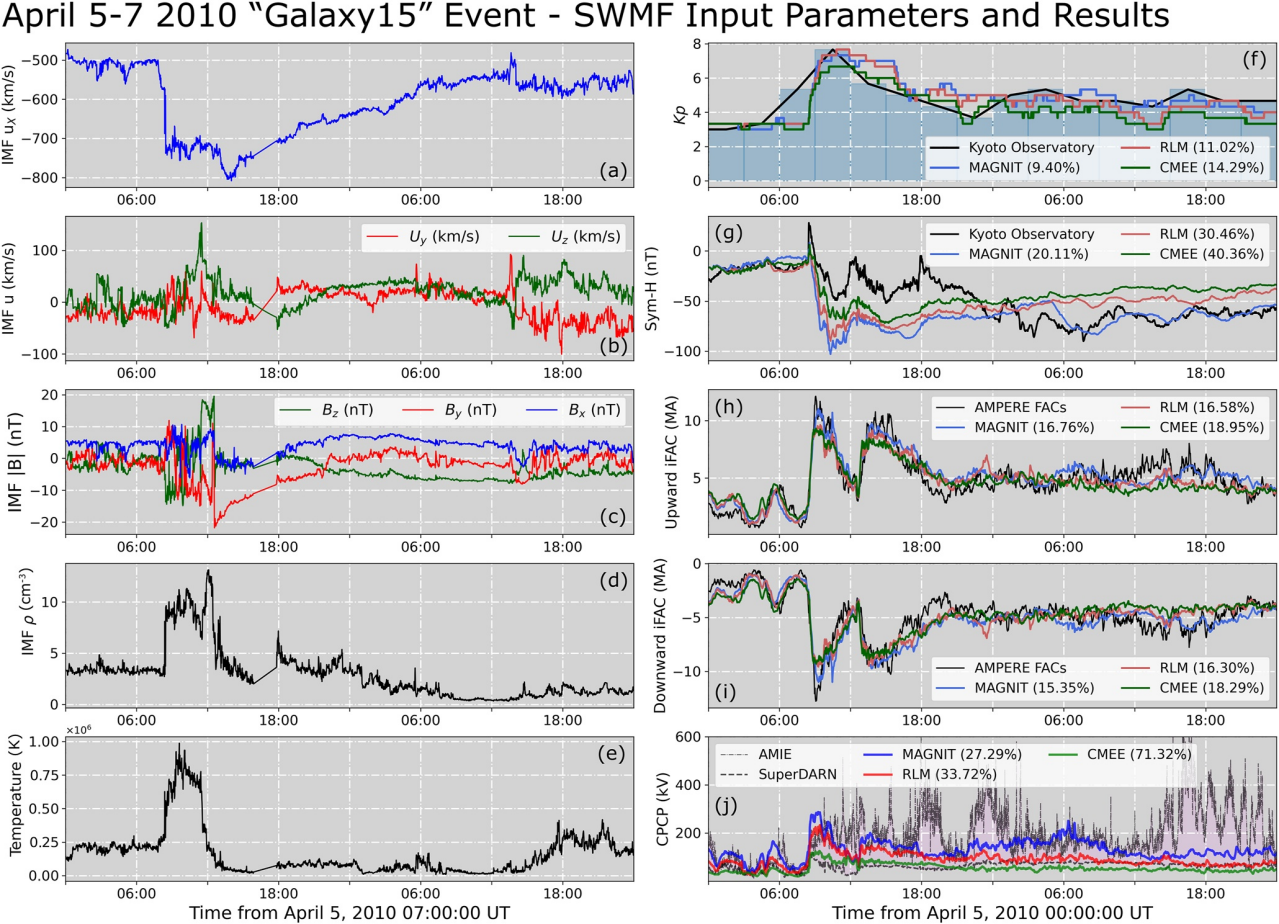
Input conditions and global results from the Space Weather Modeling Framework (SWMF) run of the *Galaxy15* event. (a) IMF *U*_*x*_, (b) *U*_*y*_ and *U*_*z*_, (c) IMF $\vec{B}$, (d) number density *ρ*, (e) particle energy. (f) *Kp*, (g) Sym‐H, (h) upward integrated field‐aligned currents (iFACs), (i) downward iFACs, and (j) cross‐polar cap potential (CPCP) simulated by MAGNetosphere‐Ionosphere‐Thermosphere (MAGNIT; in blue), Ridley Legacy Model (RLM; in red), and Conductance Model for Extreme Events (CMEE; in green) against observations (in black; Kyoto Observatory for *Kp* and Sym‐H, AMPERE FACs for iFACs). In (j), two observation‐derived sources—AMIE and SuperDARN—have been used. For (f)–(i), the MAPE between the modeled values and observations have been provided. For (j), the EP is provided.

The *Galaxy15* event is a prominent space weather interval (e.g., Allen, 2010) which has been investigated by multiple studies (e.g., Anderson et al., 2017; Chen et al., 2015; Keesee et al., 2014; Mukhopadhyay et al., 2020; Pulkkinen et al., 2013; Welling et al., 2017), and spanned from 5 April 2010 00:00 UT until 6 April 2010 23:59 UT. The event was driven by a fast interplanetary coronal mass ejection (ICME; e.g., Cane & Richardson, 2003) that caused significant dipolarization during the main phase of the storm (e.g., Connors et al., 2011), resulting in a prolonged recovery period (e.g., Möstl et al., 2010). Intense auroral activity along with multiple substorms were observed during this period (Clilverd et al., 2012; Loto'aniu et al., 2015), resulting in the event being reclassified as a supersubstorm (e.g., Nishimura et al., 2020b). Figures 5a–5e display IMF $\vec{u}$, $\vec{B}$, number density and temperature, that have been used as input conditions to drive this SMWF run. The solar plasma parameters were obtained from instruments aboard the Advanced Composition Explorer (ACE). Unlike the idealized cases in Section 3, the event was simulated without the necessity of a dedicated preconditioning period. Separate SWMF simulations were run with the empirical conductance models RLM and CMEE to compare model‐model auroral dynamics.

Panels 5f–5j show simulated space weather quantities against respective observations. The quantities compared here include the space weather indices *Kp* and Sym‐H, and ionospheric quantities iFACs and CPCP. Upward and downward iFACs have been accounted for separately in Figures 5h and 5i, respectively. MAGNIT simulations of *Kp* and Sym‐H exhibit an MAPE value of 8.73% and 19.67%, respectively. In contrast, the RLM and CMEE simulation exhibit higher values of MAPE for both *Kp* (RLM—11.02%, CMEE—14.29%) and Sym‐H (RLM—30.46%, CMEE—40.36%). Modeled predictions for both quantities show reasonable agreement throughout the event, with the exception of modeled Sym‐H during the event peak and early recovery period which was overpredicted. All models show excellent agreement with observed upward and downward iFACs. MAGNIT exhibits a ∼16% MAPE value for both upward and downward FACs, followed closely by RLM and CMEE which exhibit an MAPE of ∼16.4% and ∼18.6%, respectively. This is partly due to the numerical grid resolution of the global MHD domain, which plays a dominant role in defining FAC magnitude and structure (Mukhopadhyay et al., 2020; Ridley et al., 2010; Welling, 2019; Wiltberger et al., 2017). Comparison of the CPCP to either source of observations do not yield meaningful conclusions, as SuperDARN is clearly underestimating the CPCP while AMIE overpredicts the value (Gao, 2012; Mukhopadhyay et al., 2021b). So, it is desirable to be between the two, i.e., have a low value of EP. The modeled CPCP by the MAGNIT run had an EP value of ∼27%. In contrast, the RLM and CMEE runs had an EP of ∼33% and ∼71%, respectively. Since FACs and CPCP stand to be affected the most by a change in auroral precipitation a more exhaustive examination that quantifies modifications in the particle drift velocities and currents could be conducted, but is not the focus of the present study. Subsequent investigations will review the ionospheric electrodynamics results in greater detail.

### 4.1 Balance of Precipitation

**Figure 6:**
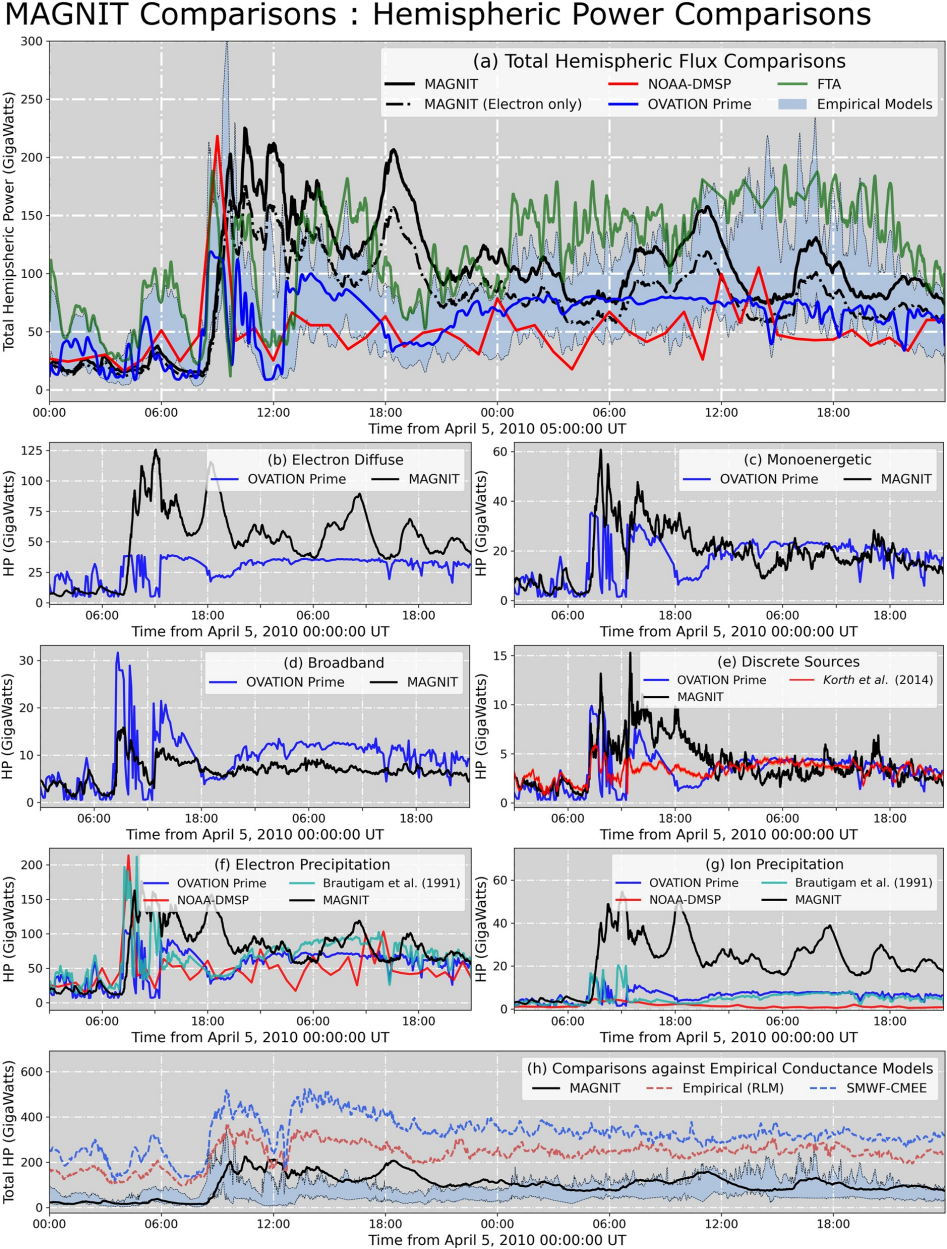
Comparison of hemispheric power estimated by MAGNetosphere‐Ionosphere‐Thermosphere (MAGNIT) against multiple observational‐derived estimates. Comparison of (a) total hemispheric power estimated by MAGNIT (black; solid line denotes all sources, dot‐dashed denotes electron sources), NOAA‐DMSP (red), OV Prime (blue), FTA (green), and four empirical models (light‐blue band). (b–d) Electron diffuse, monoenergetic, and broadband precipitation by MAGNIT (black) against OVATION Prime (deep blue). (e) Discrete precipitation in the dusk‐noon sector by MAGNIT (black) against OV Prime (blue) and Korth et al. (2014) (red). (f) Total electron precipitation, and (g) ion precipitation—MAGNIT (black) versus OV Prime (blue), NOAA‐DMSP (red) and Brautigam et al. (1991) (turquoise). (h) Hemispheric power by Ridley Legacy Model (RLM) and Conductance Model for Extreme Events (CMEE) versus MAGNIT compared with observation‐derived estimates (light‐blue band).

Figure 6 compares total and source‐wise hemispheric power from MAGNIT against multiple sources of both observations and observation‐derived estimates. Figure 6a compares MAGNIT total hemispheric power against observations by DMSP SSUSI, simulated results from OV Prime, FTA, and the four empirical models by Brautigam et al. (1991), Ahn et al. (1983), Lu et al. (1998), and Østgaard et al. (2002) (hereonafter referred to as *Brautigam91*, *Ahn83*, *Lu98*, *Ostgaard02* models, respectively). The latter four models have been bundled together to form a light‐blue band in the subplot. Solar wind inputs drive both OV Prime and *Brautigam91* models, while Kyoto‐observed AE/AL values are used to drive the FTA model and the remaining empirical models. Since several models in this plot were designed to derive hemispheric power from electrons only (e.g., FTA), the electron contribution from MAGNIT has been plotted as a dot‐dashed black line alongside the total contribution. Overall, modeled precipitation by MAGNIT exhibits reasonable agreement with both observed and derived estimates. The hemispheric power peak of 225.2 GW estimated by MAGNIT compares well against the observed peak of 218.3 GW from DMSP, albeit at different times. This is most likely due to the difference in time cadence between both data sets, with DMSP being hourly averaged and MAGNIT having a cadence of 1 min. Modeled HP are greater than OV Prime estimates during the storm peak‐time and remain larger except for a short interval during 6 April 2010 04:00 UT to 07:31 UT. Despite this, MAGNIT electron precipitation matches well against the total OV Prime estimates during the long recovery period of the storm, exhibiting an aggregate MAPE value of 24.4%. MAGNIT estimates also have good agreement with the FTA model during the main impulse of the event, but do not match during the period preceding the storm and the recovery period when FTA predicts a larger energy flux deposition. MAGNIT exhibits an 34.2% EP when compared against the range of values formed by OV Prime and FTA, denoting good agreement. Additionally, the modeled values reasonably agree against estimates from the four empirical models (EP 29%), with majority of the overprediction occurring during the main impulse of the storm.

In panel (b), MAGNIT predicts a larger value of electron diffuse power in comparison to OV Prime, resulting in a median absolute error of 19.95 GW (MAPE = 65.8%). This is expected for two reasons—(a) the finalized *α*_*s*_ values that regulate the electron diffuse flux in MAGNIT allow for a much higher diffuse flux value during moderate‐to‐extreme driving conditions (see Figure 4ii‐a), and (b) OVATION Prime is most likely underpredicting diffuse precipitation during the solar wind enhancement (e.g., Newell et al., 2014). With a maximum peak of 125.68 GW, electron diffuse precipitation accounts for a median 51.4% of the total contribution to the hemispheric power. This causes the magnitude differences between the two models, as seen in Figure 6a.

In panels (c) and (d), monoenergetic and broadband powers display good agreement with the OVATION Prime values, except during the main impulse phase and early recovery period (08:35 UT to ∼20:00 UT) of the event, when monoenergetic precipitation is overpredicted while broadband is underpredicted. However, both models predict the double peak in flux values centered around 10:45 UT and 13:30 UT. Discrete (monoenergetic + broadband) hemispheric power in the postnoon pre‐dusk (12–18 MLT) sector is compared against OV Prime and the empirical relationship given by Korth et al. (2014) in panel (e). Both models show reasonable agreement with the *Korth14* model and with each other during the latter half of the recovery period of the event. However, significant differences during the event peak are also observed: both MAGNIT and OV Prime estimate a greater discrete precipitation during this time period, compared to the *Korth14* model.

In panels (f) and (g), the total electron and ion power from DMSP, OV Prime, and *Brautigam91* indicate good agreement for electron precipitation, but show a significant disconnect in ion precipitation, with MAGNIT overpredicting the ion fluxes by over 2.5 times. This also increases the contribution of ion precipitation to the total hemispheric energy flux, to a median 22.4%. It is likely that the observed estimates are underpredicting the energy flux due to ions, since both DMSP and OV Prime do not observe particles with average energies >30 keV (e.g., Newell et al., 2009). A closer examination of ion precipitation is necessary, which will be pursued in a future study.

Finally, panel (h) compares MAGNIT HP against predictions from the empirical conductance models, RLM and CMEE, and NOAA‐DMSP, FTA, OV Prime, and the four empirical models combined (light‐blue band). RLM and CMEE predict higher fluxes than MAGNIT estimates. This is most probably because of the models' usage of empirical adjustments to increase conductances in regions of high FACs. Both RLM and CMEE overpredict the hemispheric power, with CMEE values reaching ∼500 GW during the event peak.

**Figure 7:**
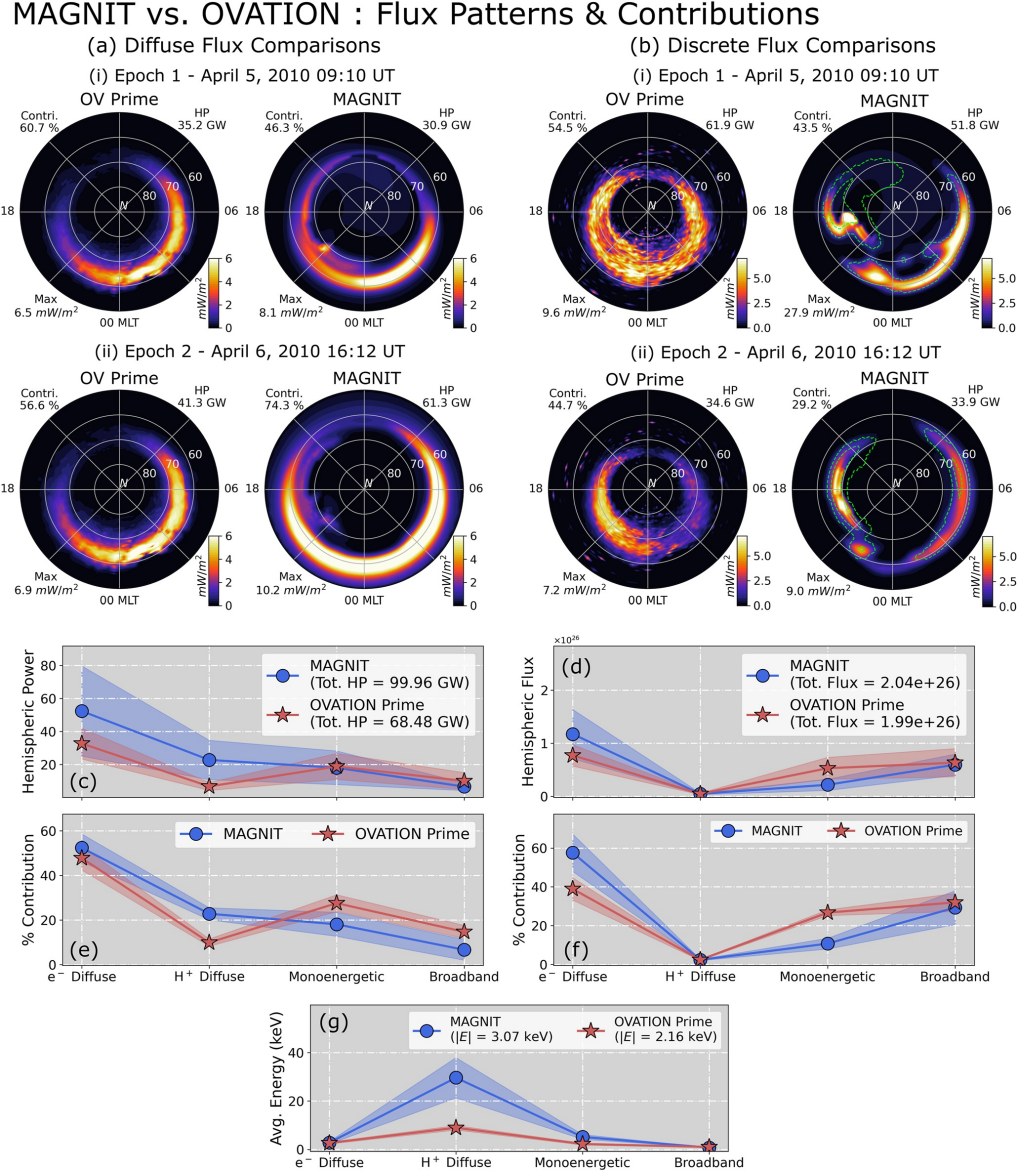
MAGNetosphere‐Ionosphere‐Thermosphere (MAGNIT) versus OVATION Prime—balance of fluxes and comparison of auroral patterns. (top) Dial‐plot comparisons of (a) diffuse energy flux and (b) discrete energy flux at two distinct epochs during the *Galaxy15* event. (bottom) Source‐wise comparison of (c) hemispheric power (in GigaWatts), (d) hemispheric number flux (in particles), (e) power contribution (in percent), (f) number flux contributions (in percent), and (g) average energy (in kilo‐electronVolt).

Figure 7 compares MAGNIT flux patterns and aggregate contributions from each source against OV Prime. In this figure, panels (a) and (b) compare auroral patterns due to diffuse (ion + electron) and discrete (monoenergetic + broadband) sources, respectively, in the Northern hemisphere for two time intervals. The first time interval, Epoch 1, is chosen at 5 April 2010 09:10 UT, during the storm onset. The second time interval, Epoch 2, is chosen at 6 April 2010 16:12 UT during the recovery period. In panel 7(a–i), modeled diffuse precipitation matches the magnitude and location of precipitation predicted by OV Prime. Diffuse sources in MAGNIT account for 37% of the total contribution at Epoch 1, indicating a larger contribution by the discrete sources during this epoch. The inverse is true for OV Prime, where diffuse sources contribute toward 60.7% of the total precipitation making them the dominant contributor. In panel 7(b–i), modeled discrete energy flux is dominated by the monoenergetic source, which is indicated by the presence of strong auroral activity in regions of upward FACs (indicated by green dotted line). These regions are discontinuous, but contain strong precipitation in both dawnward and duskward sectors. Broadband precipitation is not as strong, and therefore contributes to only 11.3% of the total energy flux. By contrast, OV Prime estimates a wide oval of energy flux spanning the 70°–60° MLat at 00 MLT, with no visible differences demarcating monoenergetic and broadband precipitation.

Figures 7a‐ii and display auroral patterns during Epoch 2. In Figure 7a‐ii, MAGNIT predicts a larger diffuse flux contribution in comparison to OV Prime, resulting in a dominant 74% of the total contribution. MAGNIT's energy flux pattern expands beyond 60° MLat, with high fluxes spanning the entire nightside. By contrast, OV Prime predicts a minimal increment in the fluxes with no noticeable expansion in the aurora. In Figure 7b‐ii, modeled discrete precipitation is still dominated by monoenergetic precipitation. But the magnitude of fluxes has reduced significantly. Diffuse fluxes from OV Prime are estimated to be more poleward than MAGNIT. Both models predict a discrete auroral power of around ∼34 GW. However, their percentage contributions are significantly different in each model, with OV Prime estimating a 45% discrete contribution, while MAGNIT predicts a much lower 29% contribution. MAGNIT observes strong monoenergetic precipitation in both dusk and dawn sectors. This is likely because the model predicts strengthened R2 FACs due to the coupling with RCM.

Figures 7c–7g compare median values of hemispheric power, number flux, and average energy for the four sources against OV Prime estimates. Subplots 7c and 7d compare the median values of hemispheric power and number flux from each source and the associated uncertainty in the data (indicated by the translucent spread around the marker). Here, the uncertainty is measured using the standard deviation in the data for each source. The figure shows that MAGNIT estimates a higher diffuse power than OV Prime. Modeled estimates for electron and ion diffuse precipitation have a median value of 52.38 and 22.82 GW, respectively. This is greater than the OV Prime estimates, which predict 32.73 GW from electron diffuse and 6.79 GW from ion precipitation. Conversely, estimates of discrete power contributions by both models are similar. While electron diffuse number fluxes are higher in MAGNIT, both models match ion number flux estimates. OV Prime predicts a higher number flux from their monoenergetic source. However, both models reasonably agree on the median number flux from broadband precipitation.

Subplots 7e and 7f compare the median contributions by each source to the total hemispheric power and number flux, respectively. The percentage contributions are computed using Equation 11. The figures show higher diffuse contributions by MAGNIT, which subsequently reduces contributions by the discrete sources. In subplot 7f, ion precipitation accounts for the smallest contributions (∼2%) to the total number flux. Electron diffuse precipitation accounts for a larger percentage of the total number flux than OV Prime prediction, while monoenergetic contribution reduces. Broadband contributions for both models show reasonable agreement with each other. Subplot 7g compares average energies of particles from all sources. MAGNIT exhibits stronger average energies for ion (29.64 keV) and monoenergetic precipitation (5.17 keV), deviating sharply from OV Prime estimates (8.96 keV for ions, 2.25 keV for monoenergetic populations) that show a 230% and 130% increase in energies of each respective source. In comparison, MAGNIT's electron diffuse (5.4% increase) and broadband (10.1% decrease) energies are similar to that predicted by OV Prime. MAGNIT estimates a more energetic ion population, compared to OV Prime. This is because of two reasons—(a) MAGNIT estimates lower ion number fluxes and higher energy fluxes and (b) OV Prime underpredicts the ion average energy (Newell et al., 2009). While higher energies are exhibited by monoenergetic precipitation as well, this is most likely due to the model's dependence on strong FACs to derive bulk of its monoenergetic precipitation, which is a function of activity.

#### 4.1.1 Comparison of Auroral Flux Patterns

**Figure 8:**
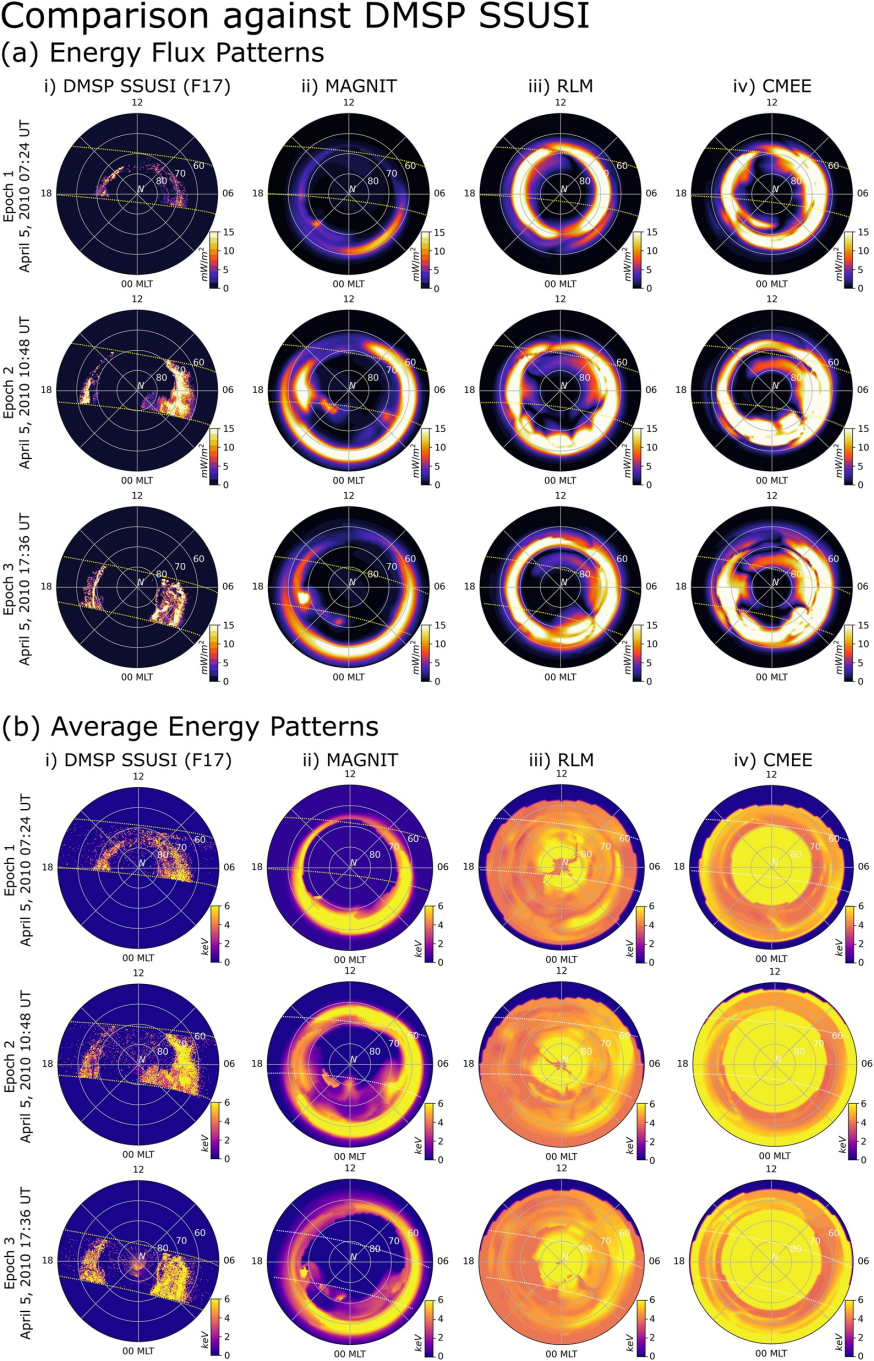
Comparison of (a) total energy flux and (b) average energy patterns in the Northern hemisphere at discrete time intervals spanning the *Galaxy15* event. (column‐wise) Dial plots display observations from (left to right) DMSP SSUSI (Column i), and simulated results from Space Weather Modeling Framework (SWMF) simulations driven using MAGNetosphere‐Ionosphere‐Thermosphere (MAGNIT; Column ii), Ridley Legacy Model (RLM; Column iii), and Conductance Model for Extreme Events (CMEE; Column iv) at three distinct time epochs. Dotted lines in each dial plot indicates span of SSUSI coverage.

Modeled auroral patterns in the Northern hemisphere have been compared against DMSP SSUSI observations in Figure 8. Figure 8a compares energy flux observations by the DMSP F17 spacecraft in the first column against simulations by (chronologically) MAGNIT, RLM, and CMEE. Each row indicates a specific time epoch. The first row indicates auroral fluxes at 5 April 2010 at 07:24 UT, before the sudden commencement of the *Galaxy15* event. During this time interval, flux pattern from DMSP shows low auroral activity with limited expansion of the oval. This observation is well reproduced by MAGNIT which match the magnitude of DMSP's energy flux. However, MAGNIT predicts an expanded auroral oval, with its equatorward boundary reaching lower 60°S MLat. Both RLM and CMEE overpredict the magnitude of energy flux, displaying a strong auroral oval with minimal auroral expansion. However, both models predict the auroral oval in similar latitude as DMSP.

Epoch 2 takes place immediately following the main impulse of the event at 10:48 UT. At this time, DMSP observations indicate a significant increase in auroral precipitation in the dawnward sector, with similar but comparatively limited enhancements in the duskward sector. The oval has expanded substantially, with the dawnward peak appearing at 67°–66° MLat and the duskward peak at 65° MLat. The dawnward flux precipitation is broader and stronger than the duskward sector, most likely caused by heightened electron diffuse precipitation. The enhancement of auroral precipitation is also captured by MAGNIT, which exhibits a higher magnitude and a distinct auroral expansion. Flux magnitudes from MAGNIT compare well against the DMSP observations matching peak regions of precipitation. However, MAGNIT overpredicts the auroral expansion by diffuse sources, estimating an equatorward dawnward peak. The flux peak in the duskward sector matches well with the DMSP observations. This is probably because this stretch of precipitation is being driven solely by monoenergetic precipitation, which follows the upward R1 FACs in the region. MAGNIT also exhibits distinct mesoscale structures poleward of the auroral oval. Both RLM and CMEE do exhibit similar flux levels as DMSP, but do not show any significant expansions in the auroral oval. This is expected from their empirical design, and is limited from expanding the aurora beyond 60° MLat (see Section 3.1 in Mukhopadhyay et al. (2020) for details).

Epoch 3 takes place during the long recovery period of the storm at 17:36 UT on 5 April 2010. During this time interval, DMSP observes a broad dawnward flux peak spanning 70°–60° MLat, and a narrow duskward peak bordering 70° MLat. Despite its capability to predict correct magnitudes, MAGNIT overpredicts the latitudinal extent of the dawnward precipitation. Auroral fluxes predicted by RLM (Column iii) and CMEE (Column iv) exhibit high energy fluxes and distinct FAC‐driven structures during these two epochs without much auroral expansion.

Figure 8b compares the average energy for the aforementioned data sets for the same epochs. Unlike part (a), only electron‐driven precipitation (electron diffuse, monoenergetic, and broadband) have been used to compute the energies in MAGNIT. This was done because the energies from ion precipitation in MAGNIT were much higher than the observational limit of DMSP (≥25 keV). DMSP observations and results from MAGNIT display significant morphological similarities. Similar to the previous part, MAGNIT exhibits an extended auroral oval in comparison to DMSP observations, but is able to match average energy magnitudes. By contrast, both RLM and CMEE are unable to produce resembling average energy patterns. The average energy patterns in both are characterized by a high energy region around the geomagnetic poles, with slight reduction in energies as one moves equatorward. This is a drawback in both these models due to their usage of FACs to compute conductances directly, and not fluxes. The average energy, therefore, is a byproduct of a reverse‐*Robinson* relationship, where the FAC‐derived conductances are converted to energies using the inverse of the relationship given in Robinson et al. (1987) (see Mukhopadhyay et al. (2020) for further details), rather than a physical manifestation of particle energies like in MAGNIT.

### 4.2 Magnetospheric Feedback

**Figure 9:**
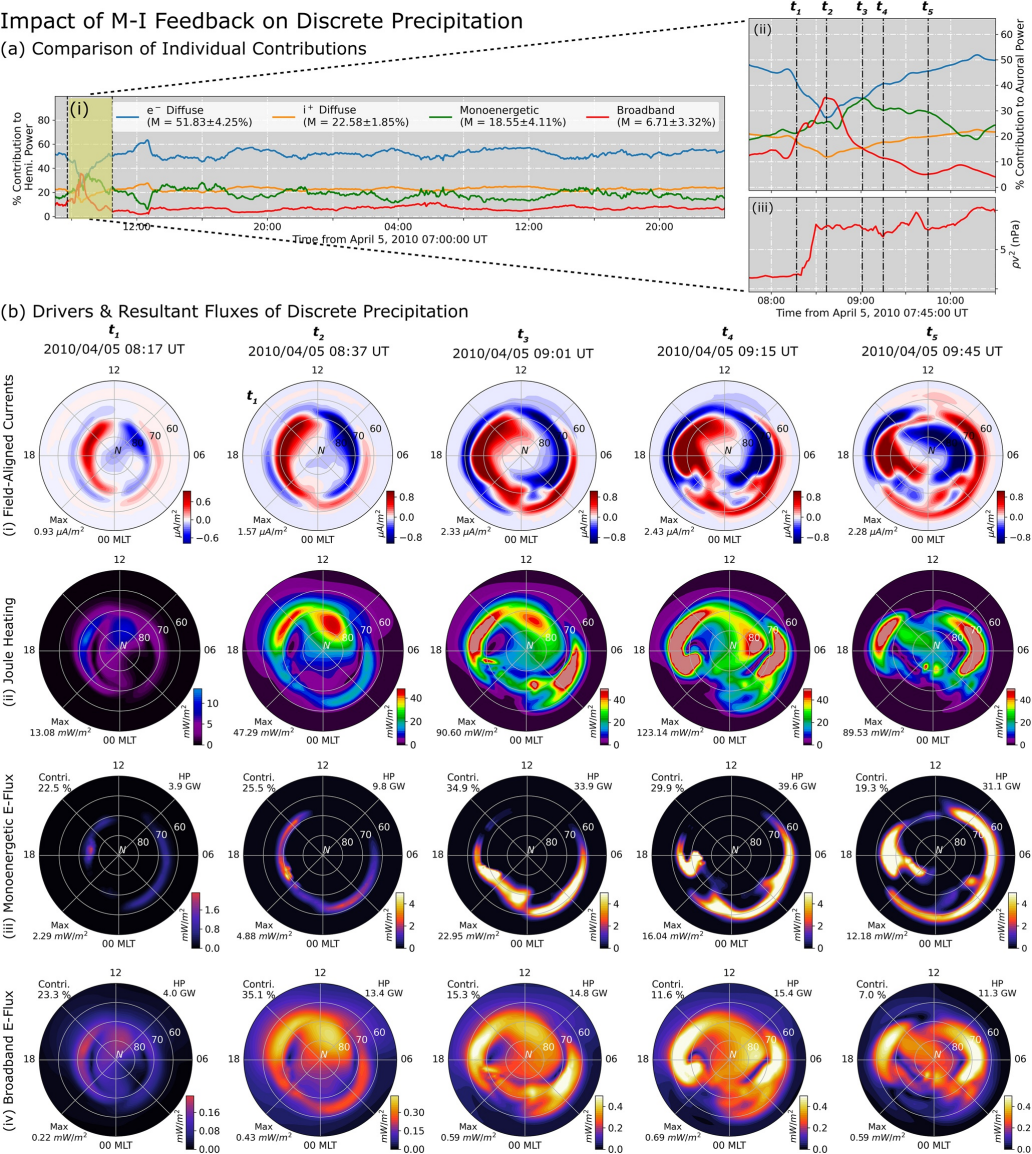
(a) Comparison of contributions by all sources during the *Galaxy15* event, with five time intervals highlighted. (b) Comparison of quantities pertaining to discrete precipitation—(i) field‐aligned currents, (ii) Joule heating, (iii) monoenergetic energy flux, and (iv) broadband energy flux. Despite the uniformity in the color bar scale, note that the color bar range for *t*_1_ is different from other times.

**Figure 10:**
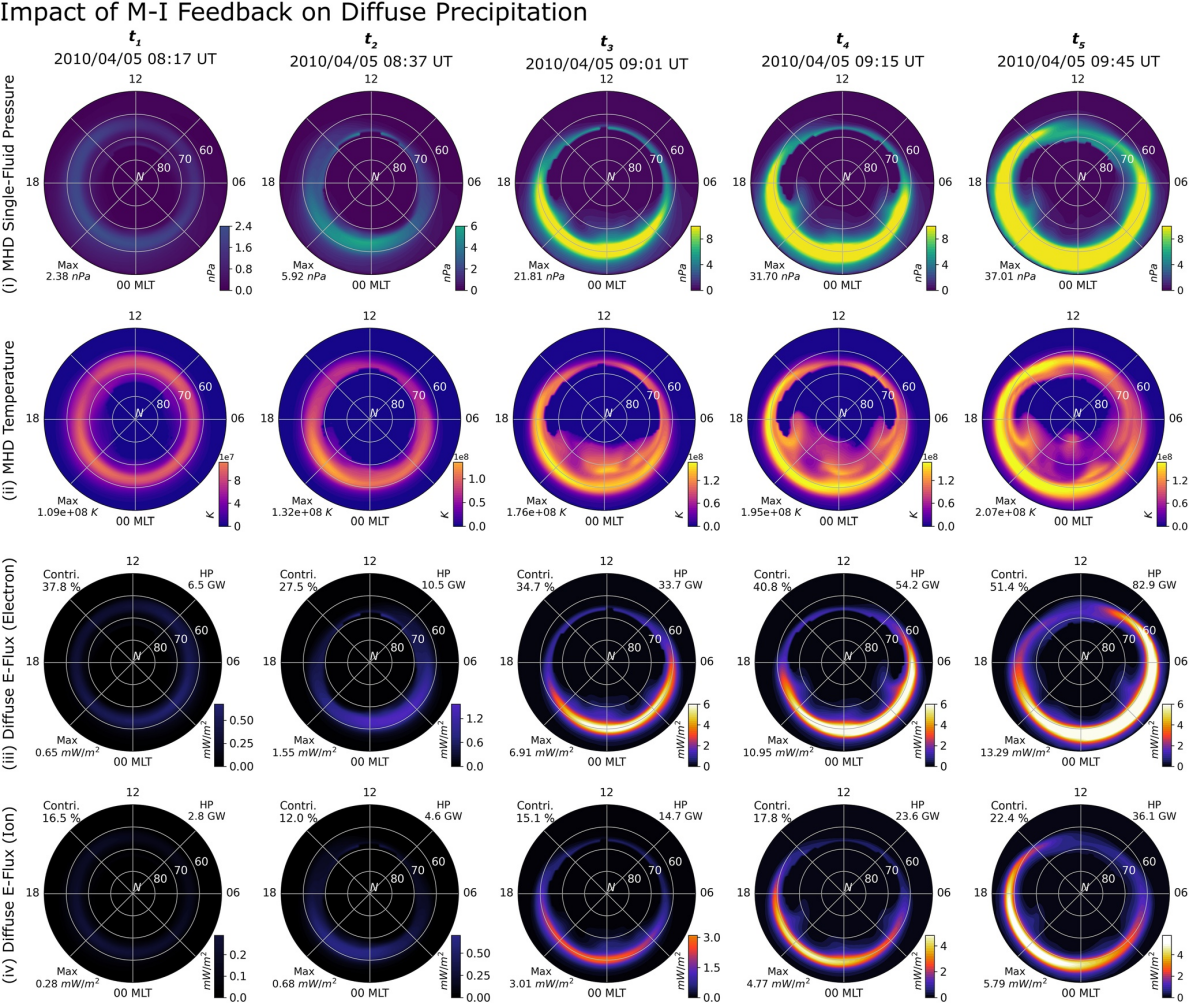
Comparison of quantities pertaining to diffuse precipitation—(i) magnetohydrodynamic (MHD) plasma pressure mapped onto the ionospheric domain, (ii) MHD plasma temperature mapped onto the ionospheric domain, (iii) electron diffuse energy flux, and (iv) ion diffuse energy flux—during each time interval identified in Figure 9a.

Due to its dynamic coupling with the magnetosphere, MAGNIT allows for better reception of magnetospheric feedback into their calculation of auroral fluxes and ionospheric conductance. Figures 9 and 10 show an example of this process. Figure 9a‐i compares the contribution by each source of precipitation during the *Galaxy15* event. For a majority of the event, the source‐wise contributions take a near‐constant value, with electron diffuse precipitation providing 51% of the total energy flux. Ion and monoenergetic precipitation contributions are each about half of the electron diffuse contribution, with a median contribution of 22.6% and 18.6% each. The remaining contribution is provided by the broadband source, which provides for a mere 6.5% of the total contribution making it the smallest source of energy flux. However, these contributions are severely distorted during the event peak where the discrete sources overtake diffuse contributions peaking at a combined contribution of 61% at 08:35 UT on 5 April 2010. The zoomed section in part (a‐ii) shows this time duration in further detail, and identifies five time intervals—08:17 (*t*_1_), 08:37 (*t*_2_), 09:01 (*t*_3_), 09:15 (*t*_4_), and 09:45 UT (*t*_5_) on 5 April 2010—to investigate the driving factors of the discrete enhancement. The time duration spans the main impulse of the *Galaxy15* event during which the solar wind ram pressure increased, as shown in part (a‐ii). The impact of magnetospheric dynamics and subsequent ionospheric interactions during a solar wind enhancement has been previously analyzed in multiple numerical studies like Yu et al. (2010), Ozturk et al. (2018), and more recently in Welling et al. (2021). In this study, we investigate these dynamics in regards to changes in auroral precipitation.

In Figure 9b, the response of four quantities—(row‐wise) FACs, Joule Heating, monoenergetic energy flux, and broadband energy flux—have been displayed for the five time intervals. At *t*_1_, FAC patterns follow the standard southward *B*_*z*_ model with strong R1 FACs and relatively weaker R2 FACs, and are symmetric about the noon‐midnight meridian with an integrated total current of 4.51 MA. Joule heating in the second row is computed using the Pedersen conductivity and electric field (e.g., Rastätter et al., 2016), and appear in the same region as the FACs exhibiting a maximum flux of 13.08 mW/m^2^. Since the model is not coupled to a dedicated ionosphere‐thermosphere model, the Joule heating does not incorporate contributions from the neutral wind. The third and fourth rows display monoenergetic and broadband energy flux. Both FACs and Joule heating are the dominant drivers of these two respective sources. Monoenergetic precipitation, which follows the KFL relationship, is strong only in regions of upward FACs and accounts for 22.5% of the total hemispheric power. Similarly, broadband enhancements are derived as an empirical function of the Joule heating, and contributes to 23.3% of the total power.

Time interval *t*_2_ takes place 7 min after the main impulse of the event that leads to ram pressure enhancement in the solar wind. This interval is readily characterized by the strengthening of FACs and Joule heating patterns. FACs are still symmetric about the noon‐midnight meridian, but exhibit significant enhancement in R1 currents and stronger R2 currents. *t*_2_ observes a doubling of integrated total current to 10.1 MA. A significant dayside peak emerges in the Joule heating pattern, with the maximum flux surging to 47.3 mW/m^2^. Correspondingly, the discrete fluxes react to these changes with an enhancement in precipitation. Monoenergetic flux rises from 3.9 GW at *t*_1_ to 9.8 GW at *t*_2_, with a significant contribution in the R2 FACs (dawnward) sector in addition to the upward R1 FACs in the dusk sector. Broadband precipitation increases by 3.35 times, becoming the dominant contributor to auroral precipitation. Discrete contributions account for 60.5% of the total precipitation, in contrast with contributions during the rest of the event.

During *t*_3_ (24 min after *t*_2_, 31 min after the pressure enhancement), R1 FACs start expanding in addition to becoming more enhanced in magnitude. The dominant peaks expand beyond 70° MLat, resulting in a further expansion of the R2 currents into the upper 50° MLat. The FAC pattern increasingly becomes asymmetric across the noon‐midnight meridian with small‐scale structures connecting the R1 and R2 FACs appearing in the nightside. Two dominant peaks arise in the Joule heating in the dusk and dawnward flanks, with the duskward peak appearing in the dayside region of the peak at *t*_2_. The dawnward peak appears in the nightside, with the pattern being symmetric about the 10–22 MLT meridian. The enhancement in monoenergetic precipitation is characterized by the strengthening of FACs, and accounts for 34.9% of the total precipitation. Despite the strengthening of FACs in both the dayside and nightside alike, the monoenergetic population is mostly concentrated on the nightside. This is most likely because the diffuse number flux is low on the dayside, i.e., there is insufficient particles in the dayside to accelerate. Broadband precipitation is concentrated in the Joule heating peaks in the two flanks of the Northern hemisphere. However, due to strengthening of the other sources of precipitation, the contribution by broadband precipitation reduces to 15.3%.

Time intervals *t*_4_ and *t*_5_ display conditions at 09:15 UT (14 min after *t*_3_, 45 min after the pressure enhancement) and 09:45 UT (44 min after *t*_3_, 1.25 hr after the pressure enhancement). During these time intervals, the FAC expansion observed during *t*_3_ reaches its furthest point and plateaus, with R1 current peaks extending from the upper 70° MLat to mid‐60° MLat while R2 currents reach down to mid‐50° MLat. Both R1 and R2 FACs are further strengthened and become increasingly asymmetrical. Joule heating patterns have a similar two‐peak configuration as seen at *t*_3_. The peaks lie in the same region as the upward FAC peaks, albeit being much broader than R2 peaks in the dawnward sector. Correspondingly, monoenergetic precipitation increases along with FAC enhancements and expansion, resulting in a total hemispheric power of 39.6 GW during *t*_4_ and 31.1 GW during *t*_5_. Due to the increase in diffuse flux during these times in the dayside sector, monoenergetic precipitation extends into the dayside in both dawnward and duskward sectors. Broadband precipitation follows the two‐peak configuration of the Joule heating pattern, and results in a total power of 15.4 GW at *t*_4_ and 11.3 GW at *t*_5_. Contributions by discrete sources to the total precipitation drops down from 60.5% during *t*_3_ to 41.5% during *t*_4_ and 26.3% during *t*_5_ of the total precipitation, indicating significant enhancement in diffuse precipitation.

Figure 10 describes the impact of the solar wind pressure enhancement on diffuse precipitation. The MHD single‐fluid pressure and temperature have been mapped to the ionospheric grid for clarity, and have been displayed in the first and second rows, respectively, for the same time intervals as in Figure 9b. Since both these quantities drive diffuse precipitation in MAGNIT, the energy flux from electron and ion precipitation have been plotted in the third and fourth rows. Comparison of the first row across the five time intervals indicates a steady enhancement in the nightside pressure. This enhancement in the pressure also brings the pressure peaks in nightside closer to Earth, resulting in an equatorward expansion of the nightside peak on the ionospheric grid. The buildup in nightside pressure is gradual, as can be seen during *t*_1_, *t*_2_, and *t*_3_, and is not slower than the ramp‐up in FACs. This is expected, since asymmetric currents that drive FACs in the system buildup much quicker than symmetric pressure used here (e.g., Liemohn et al., 2015). Before the solar wind enhancement, the nightside pressure at *t*_1_ is expected to be less. Postram pressure enhancement, at *t*_2_, the increase in pressure is demarcated by the formation of an oval with a peak in the midnight‐dusk sector. However, the pressure increases steadily over the next 24 min, culminating in a sharp nightside peak with a band spanning the auroral region. The pressure peak expands equatorward from their previous location at *t*_2_, and quadruples in magnitude. Intervals *t*_4_ and *t*_5_ exhibit further enhancement and expansion of the nightside pressure peak. At *t*_5_, the pressure peak moves further equatorward reaching the lower 50° MLat. A similar buildup of energy is observed in the second row, when comparing particle temperatures. Contrary to the pressure distribution, the MHD temperature distributions during *t*_3_, *t*_4_, and *t*_5_ exhibit strong mesoscale dynamics poleward of the auroral peak formed through the mapping. The dynamic structures in the temperature distribution change with the March in time, occasionally strengthening the peaks in the oval region, like in *t*_3_.

The electron and ion diffuse energy fluxes are compared in the last two rows of Figure 10. During *t*_1_, electron and ion diffuse precipitation account for 37.8% and 16.5% of the total energy flux, respectively, with the ion precipitation accounting for the lowest contribution. However, energy fluxes from both these sources are comparatively lower and exhibit a diffuse band of precipitation centered around the nightside pressure peak (for ions) and its mirrored location (for electrons). With the solar wind pressure enhancement, the fractional contribution of the diffuse sources reduces, since discrete fluxes enhance faster resulting in the electron diffuse sources to contribute to 27.5% of the total flux during *t*_2_. At *t*_3_, the pressure peak in the nightside is sufficiently strong to raise the diffuse hemispheric power to 48.4 GW, accounting for nearly half of the total precipitation. The electron diffuse precipitation at *t*_3_ is characterized by a sharp peak in the midnight‐dawnward sector, with the auroral oval expanding beyond 60° MLat. The ion precipitation mirrors this configuration resulting in a total hemispheric power of 14.7 GW. During *t*_4_ and *t*_5_, both diffuse precipitations enhance in magnitude and expand further equatorward. Electron diffuse precipitation becomes the dominant contributor to the total precipitation, accounting for 51.4% of the total precipitation by *t*_5_. Contributions by ion precipitation are similarly enhanced, contributing 22.4% of the total precipitation greater than either discrete sources.

### 4.3 Impact on Ionospheric Conductance

**Figure 11:**
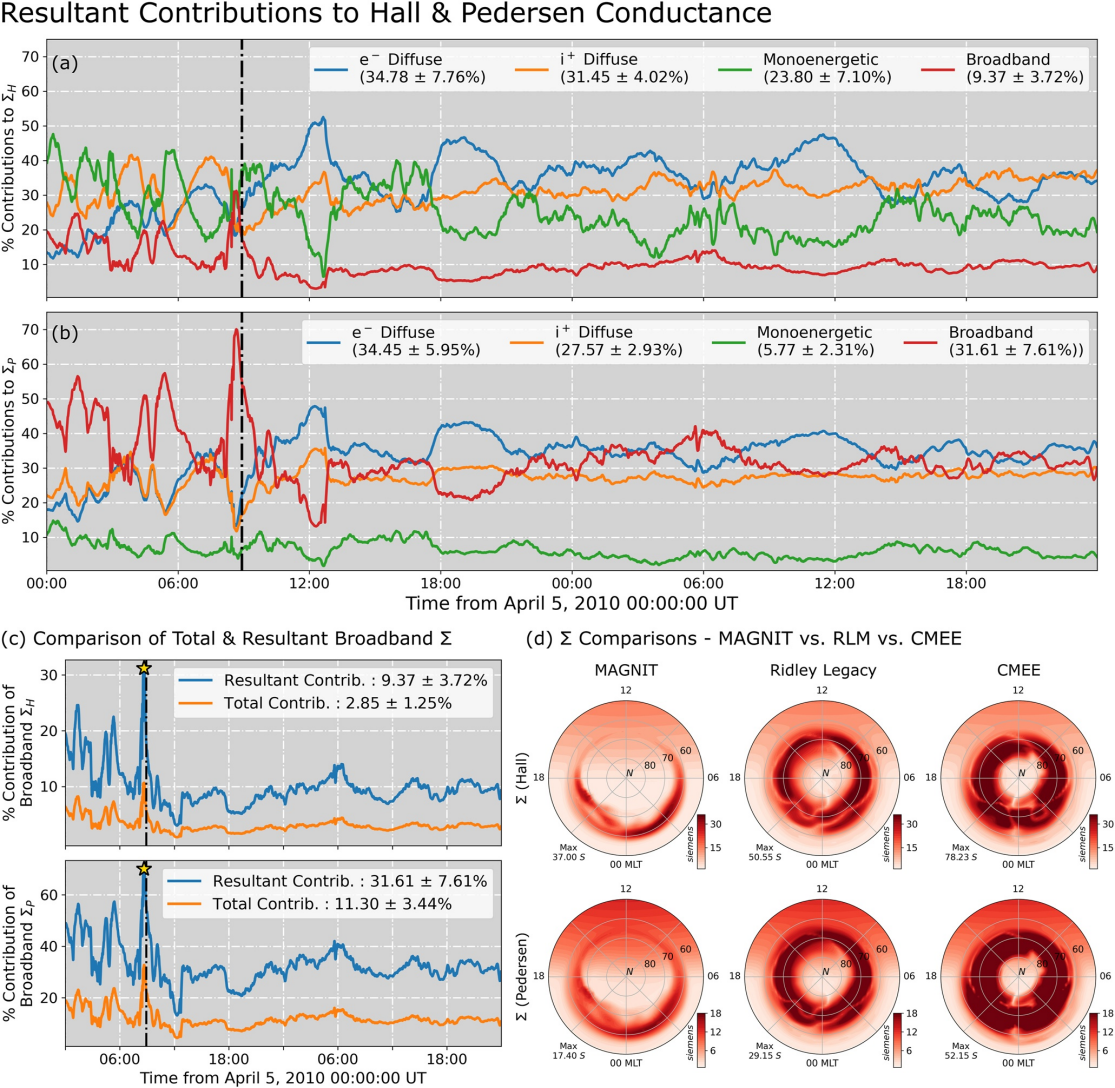
Individual contributions to perpendicular conductance—(a) comparison of individual resultant contributions by each source of precipitation to the Hall conductance and (b) Pedersen conductance. (c) Comparison of total and resultant contributions by broadband precipitation to Hall and Pedersen conductances. (d) Polar patterns of total conductance on 5 April 2010 at 08:55 UT, simulated using MAGNetosphere‐Ionosphere‐Thermosphere (MAGNIT), Ridley Legacy Model (RLM), and Conductance Model for Extreme Events (CMEE).

Figure 11 presents detailed comparisons of contributions to the ionospheric conductance by each source of precipitation. Figures 11a and 11b present individual contributions from each source to the Hall and Pedersen conductances during the *Galaxy15* event. These values are calculated by Equation 12 for monoenergetic, electron, and ion diffuse contributions, and by Equation 13 for broadband contributions. Electron diffuse precipitation is the largest contributor to both Hall and Pedersen conductances, accounting for a contribution of ∼34% to both types of conductance. This is closely followed by ion precipitation which accounts for 31% of the Hall conductance, while being the third largest source of Pedersen conductance. Monoenergetic precipitation accounts for 23.3% of the Hall conductance. However, this proportion dwindles down to a mere 5.77% contribution to the Pedersen conductance. The opposite is true for broadband precipitation which accounts for 9.4% of the Hall conductance, and 31.4% of the Pedersen conductance making it the second‐largest source to Pedersen conductance after electron diffuse precipitation. The disparity in contributions for the two discrete sources could be explained by the nature of these flux populations and how they interact with the *Robinson* relation when converted into conductance. Monoenergetic precipitation is generally the more energetic source of electron precipitation. This leads to a higher Hall conductance value through the Robinson relationship, as the Hall conductance term is directly proportional to the average energy ${\bar{E}}^{0.85}$. Broadband precipitation is the least energetic population, and therefore possesses a low average energy but high number flux. Since the *Robinson* relationship defines the Pedersen conductance as being directly proportional to the square root of the energy flux, this increases the contribution of the broadband source. Furthermore, the linear addition of the broadband contribution in Equation 10 significantly raises its resultant contribution, leading to aforementioned disparity.

The enhancement in the broadband contribution is further explained in Figure 11c. Here, the contribution by broadband conductance to the total auroral conductance is computed by two methods—(a) as a fraction of the total conductance (total contribution; computed using Equation 11) and (b) actual contribution due to its linear sum (Resultant contribution; computed using Equation 13). Comparison of both contributions indicates that the resultant contribution of broadband conductance to the total auroral conductance is much higher than expected. The resultant contribution of the broadband source is nearly ∼3 times their numerical value due to the linear addition. Driving conditions during the early phase of the event causes fluctuating contributions that can contribute to nearly 71% of the total auroral Pedersen conductance. The heightened contribution of broadband precipitation naturally postulates an important role played by this source of precipitation in ionospheric electrodynamics.

Figure 11d compares dial plots of Hall and Pedersen conductance patterns simulated by MAGNIT, RLM, and CMEE. The conductance patterns are simulated at 08:55 UT on 5 April 2010. Both RLM and CMEE provide higher conductances in the auroral region in comparison to MAGNIT. In MAGNIT, the auroral conductance is mostly concentrated on the night, with minor flanks of the dawnward and duskward fluxes reaching into the dayside sector. By contrast, significant dayside precipitation is visible in both RLM and CMEE, which exhibit a thicker auroral oval. Despite this, MAGNIT exhibits a more expanded auroral oval, with strong contributions by the monoenergetic and electron diffuse sources. RLM follows the FAC pattern closely, creating a strong R1 FAC systems with discontinuities in the conductance where the FACs change polarity. This is similar in CMEE, where the magnitude of conductance is much higher leading to a more dynamic auroral oval featuring several FAC‐driven structures in polar regions.

## 5 Discussion

The introduction of MAGNIT to the SWMF environment is a significant step forward toward computing realistic precipitation in the global model. First, the incorporation of advanced coupling mechanisms between BATS‐R‐US and RIM allow for the computation of multiple sources. These mechanisms establish a solid roadmap for future advancements in computing fluxes and conductances in RIM, that may involve further physics‐based couplings with MHD or ring current models. Second, computation of precipitation in MAGNIT is far more realistic in comparison to its empirical predecessors. Both RLM and CMEE estimate higher energy fluxes and incorrect average energies in the auroral region. MAGNIT outperforms both models when predicting energy flux (as shown in Figure 6h), and results in relatively lower but sharper conductance contributions in the auroral region. The use of MHD variables to compute auroral fluxes also means a flexible activity‐driven oval expansion, eradicating the problem observed in the empirical models during active periods (see Section 3.1 in Mukhopadhyay et al. (2020)). Third, the ability to compute four individual sources of precipitation allows for the quantification of source‐wise contributions to other ionospheric variables. Investigations quantifying the individual impacts of each source on FACs, ionospheric potential and E‐fields will soon be presented in a subsequent manuscript. Fourth, this adds to SWMF's capability to quantify the dependence of space weather results on distinct auroral precipitation types (Vandegriff et al., 2020, 2021). Finally, results simulated with MAGNIT show good agreement with observations and state‐of‐the‐art empirical models. The magnitude of auroral precipitation agrees well with both DMSP‐NOAA and OV Prime. Further comparisons of more events are underway, and will be presented in future investigations.

Note that there are several modeling caveats to this study. The auroral flux patterns formed in MAGNIT simulations, as seen in Figures 7a and 8b, show that the expanded oval of the diffuse aurora is more equatorward than those in observations. In both OV Prime (in reference to Figure 3) and DMSP (in reference to Figure 8), the dawnside peak of auroral precipitation lies in the middle of the 60–70° MLat range, while the dawnward sector in MAGNIT is characterized by a much more expanded diffuse oval peaking between 55° and 65°. This is most likely because MAGNIT computes diffuse precipitation using a Maxwellian distribution computed from the single‐fluid plasma temperature provided by BATS‐R‐US. This is problematic, as the MHD temperature is that of ions, and is converted into electron temperature by assuming a 1:5 ratio between electrons and ions. Ion temperature and pressure in global MHD is generally closer to Earth, since plasma sheet ions penetrate deeper into the inner magnetosphere than electrons (e.g., Ejiri et al., 1980; Ganushkina et al., 2000), especially during active periods (Gkioulidou et al., 2015; Yang et al., 2011). When mapped onto an ionospheric grid, these values are situated more equatorward of electron‐associated pressure and temperature values. An example of this can be seen in Jordanova et al. (2012) where the difference between ion and electron flux patterns in the nightside shows 15 keV ion flux peaks being closer to Earth than for electrons.

Usage of a dedicated electron temperature in the MHD equations would result in a nightside pressure peak that is farther away from the ion pressure peak. This would automatically result in an electron precipitation pattern that is more poleward than current estimates. The dedicated computation of multifluid (e.g., Glocer et al., 2009) and multispecies (e.g., Welling & Ridley, 2010) MHD pressure and temperature is possible through BATS‐R‐US. An alternative solution to improve diffuse calculations is to use particle fluxes computed by the ring current model. Yu et al. (2016) demonstrated the computation of electron fluxes using a coupled version of SWMF with the RAM‐SCB model. An extension to this work was performed by Perlongo et al. (2017), where the RAM‐SCB model was driven by empirical waves to compute ionospheric conductance in GITM. More recently, work by Lin et al. (2019, 2021) have sought to use RCM‐derived electron diffuse fluxes and MHD‐computed monoenergetic fluxes to compute the resultant auroral precipitation in the LFM MHD model. The use of a ring current model also results in more accurate computation of fluxes, since the models are able to account for pitch angle distributions. Recent work by Kang et al. (2019) has shown the incorporation of wave‐induced diffuse precipitation with a dedicated coupling between the ring current model CIMI and BATS‐R‐US. Work toward incorporating such solutions to provide MAGNIT with a more accurate value of diffuse precipitation will increase our physical understanding of auroral dynamics, and is planned for future work.

Computing monoenergetic fluxes using the KFL relationship requires an accurate knowledge of the global magnetic field, especially that in the source region and at the ionosphere. At present, MAGNIT assumes a dipole configuration (resulting in a latitudinally varying magnetic mirror ratio), with the source region for precipitating electrons in the plasma sheet (e.g., Yu et al., 2016). This simplifies the exact contribution by this source; the source region of precipitating plasma is located higher than the equatorial plasma sheet (Hatch et al., 2019). While this assumption does not significantly impact the resultant fluxes from the model (most likely due to the high value of *α*_*s*_), it does impact the generation of mesoscale structures in the poleward and equatorward boundaries which could further impact ionospheric electrodynamics. The computation of a realistic magnetic field is possible through the field line tracing component of SWMF, and is being currently implemented to provide a realistic magnetic field in the calculation of monoenergetic precipitation in our model. Results pertaining to these developments will be presented in future investigative studies.

The median values of *α*_*s*_ listed in Table 2 have been used to regulate fluxes in MAGNIT. This has significant disadvantages, since particle scattering rates (which *α*_*s*_ emulates for diffuse and monoenergetic fluxes) are prone to modifications in the geomagnetic field leading to variable precipitation in different sectors. Furthermore, quantities like FACs and plasma pressure are highly dependent on numerical grid resolution (e.g., Haiducek et al., 2017; Ridley et al., 2010) and the inclusion of a dedicated ring current model (e.g., De Zeeuw et al., 2004). Changes in either of these factors would result in modifications in *α*_*s*_ values for variable driving conditions. A remedy to such an issue would be to use an activity‐driven MLT‐wide map of *α*_*s*_, which tweaks auroral fluxes in regions of interest for a given upstream driving condition. In retrospect, attempts to estimate physics‐based precipitation using extended couplings to the MHD model or the inner magnetospheric model is a more worthwhile solution, since it can provide a realistic physics‐derived reasoning for flux outputs.

The computation of the final value of ionospheric conductance is contentious, as it tends to elevate the contribution of broadband precipitation significantly. As shown in Figure 11c, the resultant contribution of broadband‐driven Pedersen conductance jumps to a median 31% of the total. The physics associated with the summation of conductance sources (Wallis & Budzinski, 1981) is challenging to solve in a 2‐D ionosphere. In this work, we have followed the example of Zhang et al. (2015) when including broadband precipitation. They state that the conductance due to broadband precipitation adds to the bottomside F‐layer of the ionosphere, instead of the E‐layer, where the dominant conductivity peaks in the Hall and Pedersen conductances are found (e.g., Schunk & Nagy, 2009). This is most likely because broadband precipitation exhibits lower average energy, resulting in its deposition at an upper layer. To identify this difference in altitude, the broadband‐driven conductance was added linearly to the net conductance, as was done by Zhang et al. (2015). To truly solve this issue, a dedicated coupling to a 3D ionosphere‐thermosphere solver is necessary. Work by Burleigh et al. (2019) has introduced novel couplings between the geospace version of the SWMF with GITM, specifically when it comes to computing ionospheric conductance realistically. The incorporation of this approach with MAGNIT‐driven flux computations leads to a more realistic ionospheric feedback. Future studies by authors will feature the combination and planned usage of this modeling approach prominently in studying terrestrial (and planetary) plasma dynamics during extreme events.

## 6 Summary

A novel modeling approach was developed and used to study the 5–7 April *Galaxy15* event. The model uses mapped MHD pressure and density as inputs to derive four sources of precipitation—electron diffuse, ion, monoenergetic, and broadband. Precipitation from each source is regulated using empirical multipliers that ultimately define the balance between each source. The investigation of this modeling study focused on quantifying the contribution of each source of precipitation during the *Galaxy15* event, and found important results through the comparison of auroral fluxes, average energy, and ionospheric conductance to observations and empirical modeling techniques. Important findings are summarized in the following:

1. Electron diffuse precipitation is the dominant source of auroral precipitation during the *Galaxy15* event, accounting for a median 52% of the total hemispheric power. Ion diffuse and monoenergetic precipitation act as secondary sources of auroral precipitation, accounting for 22% and 19% of the total power. Broadband precipitation contributes for 7%, making it the smallest contributor to hemispheric power.
2. Auroral fluxes were converted into ionospheric conductance using empirical relationships, and used in the two‐way coupling between RIM and BATS‐R‐US. The individual contributions of each source to the total conductance were quantified. Despite its small contribution to hemispheric power, the linear addition of broadband‐driven conductances result in a 31% contribution to the Pedersen conductance, and 9% to the Hall conductance.
3. Despite the dominance by diffuse precipitation, discrete precipitation accounted for up to 61% of the total hemispheric flux during the main impulse of the *Galaxy15* event.
4. Comparison of hemispheric power against DMSP and OV Prime exhibit a higher ion energy flux in MAGNIT estimates, indicating higher average energy of ions in the model results relative to the observations. The electron diffuse precipitation is also larger than the value predicted by OV Prime.
5. Due to usage of a single‐fluid ion pressure and density to derive electron diffuse precipitation, MAGNIT places the dawnward peak too far equatorward, but gets the monoenergetic peak in the dusk sector at the correct location.
6. By basing particle precipitation calculations on MHD state variables that are more tied to the drivers of ion and electron precipitation instead of only FACs, both the computation and understanding of feedback in the nonlinear M‐I system are improved by this modeling approach.

## Data Availability

Simulation data for all runs have been made available via UM Deep Blue Data archive, accessible through https://doi.org/10.7302/rtmh-x457.
